# Lateral cephalometric parameters among Arab skeletal classes II and III patients and applying machine learning models

**DOI:** 10.1007/s00784-024-05900-2

**Published:** 2024-09-03

**Authors:** Kareem Midlej, Nezar Watted, Obaida Awadi, Samir Masarwa, Iqbal M. Lone, Osayd Zohud, Eva Paddenberg, Sebastian Krohn, Erika Kuchler, Peter Proff, Fuad A. Iraqi

**Affiliations:** 1 Department of Clinical Microbiology and Immunology, Sackler Faculty of Medicine, University of Tel-Aviv, Tel-Aviv, 6997801 Israel; 2 Center for Dentistry Research and Aesthetics, Jatt, 4491800 Israel; 3 Gathering for Prosperity Initiative, Jatt, 4491800 Israel; 4 Department of Orthodontics, Faculty of Dentistry, Arab America University, Jenin, PNA Palestine; 5 Department of Orthodontics, University Hospital of Regensburg, University of Regensburg, 93047 Regensburg, Germany; 6 Department of Orthodontics, University of Bonn, D-53111 Bonn, Germany

**Keywords:** Malocclusion, Class II, Class III, Cephalometric parameters, Disease classification

## Abstract

**Background:**

The World Health Organization considers malocclusion one of the most essential oral health problems. This disease influences various aspects of patients’ health and well-being. Therefore, making it easier and more accurate to understand and diagnose patients with skeletal malocclusions is necessary.

**Objectives:**

The main aim of this research was the establishment of machine learning models to correctly classify individual Arab patients, being citizens of Israel, as skeletal class II or III. Secondary outcomes of the study included comparing cephalometric parameters between patients with skeletal class II and III and between age and gender-specific subgroups, an analysis of the correlation of various cephalometric variables, and principal component analysis in skeletal class diagnosis.

**Methods:**

This quantitative, observational study is based on data from the Orthodontic Center, Jatt, Israel. The experimental data consisted of the coded records of 502 Arab patients diagnosed as Class II or III according to the Calculated_ANB. This parameter was defined as the difference between the measured ANB angle and the individualized ANB of Panagiotidis and Witt. In this observational study, we focused on the primary aim, i.e., the establishment of machine learning models for the correct classification of skeletal class II and III in a group of Arab orthodontic patients. For this purpose, various ML models and input data was tested after identifying the most relevant parameters by conducting a principal component analysis. As secondary outcomes this study compared the cephalometric parameters and analyzed their correlations between skeletal class II and III as well as between gender and age specific subgroups.

**Results:**

Comparison of the two groups demonstrated significant differences between skeletal class II and class III patients. This was shown for the parameters NL-NSL angle, PFH/AFH ratio, SNA angle, SNB angle, SN-Ba angle. SN-Pg angle, and ML-NSL angle in skeletal class III patients, and for S-N (mm) in skeletal class II patients. In skeletal class II and skeletal class III patients, the results showed that the Calculated_ANB correlated well with many other cephalometric parameters. With the help of the Principal Component Analysis (PCA), it was possible to explain about 71% of the variation between the first two PCs. Finally, applying the stepwise forward Machine Learning models, it could be demonstrated that the model works only with the parameters Wits appraisal and SNB angle was able to predict the allocation of patients to either skeletal class II or III with an accuracy of 0.95, compared to a value of 0.99 when all parameters were used (“general model”).

**Conclusion:**

There is a significant relationship between many cephalometric parameters within the different groups of gender and age. This study highlights the high accuracy and power of Wits appraisal and the SNB angle in evaluating the classification of orthodontic malocclusion.

**Supplementary Information:**

The online version contains supplementary material available at 10.1007/s00784-024-05900-2.

## Introduction

The World Health Organization (WHO) considers malocclusion one of the most essential oral health problems after caries and periodontal disease [1, 2]. **S**keletal **c**lass **II m**alocclusion (**SCIIMO**) accounts for over one-third of all malocclusions worldwide and is more frequent in Caucasians [2]. In contrast, **S**keletal **c**lass **III m**alocclusion (**SCIIIMO**) is the least frequent, with a mean frequency of 7.2%. Reports have shown that the countries with the lowest prevalence index were Italy (1.6%), Nigeria (1.6%), and Jordan (1.4%) [3, 4]. Skeletal Class II is usually defined as a change in the relationship between the two jaw (Maxilla and Mandible) bases, with a protruded position of the upper jaw to the mandible (maxillary protrusion or maxillary prognathism) or a mandibular retrusion (mandibular retrognathism) or a combination of both situations [2, 5]. Skeletal Class III is usually defined as a change of the relationship between the two jaw (Maxilla and Mandible) bases, with the mandible protruding (mandibular prognathism) from the upper jaw or a retrusion of the upper jaw (maxillary retrognathism (midface) or a combination of both conditions [1, 3].

Previous studies defined Skeletal malocclusion as a complicated disorder produced by the combination of multiple factors, such as genetics, environment, ethnic factors, nonnutritive sucking habits, impaired nasal breathing, and functional atrophy of the maxilla [3, 6–9].

The diagnosis of skeletal deformities depends on accurate measurement of distances, planes, and angles between landmarks of hard and soft tissues using lateral cephalogram and cone-beam computed tomography (CBCT), which are then traced to assess the craniofacial relationships of the teeth to the jaws and the jaws to the rest of the facial skeleton to aid in orthodontic diagnosis [10, 11]. Many approaches are applied to diagnose skeletal malocclusion. According to Steiner [12], analysis was made by the ANB angle (SNA - SNB) for classes II and III as follows - ANB angle with values > 4° = Skeletal Class II, and ANB angle with values < 0° = Skeletal Class III.

According to Jacobson [13], the Wits appraisal and ANB angle define the relation between the two jaws, with an advantage of the ‘Wits” appraisal over that of the conventional ANB angle, with a more reliable indication of the extent or severity of anteroposterior skeletal disharmony of the jaws [13]. In the following years, various studies demonstrated equations that consider the individual properties of the ANB angle. In 1977, Panagiotidis and Witt [14] showed an equation for the individualized ANB individual as ANB_ind_ = (− 35.16 + 0.4 · SNA + 0.2 · ML-NSL). Järvinen estimated the individualized ANB by applying another formula as ANB_ind_ = (ANB − (0.472 × SNA) + (0.204 × SN-MP) − 43.386) [15], and also established a regression equation to individualize the norm of the Wits appraisal as: Wits = (1.636 × ANB – 0.512 × NSL/OL – 0.830 × SNA + 71.36) [16]. In a separate study that was done on the Chinese population, the derived equations were: ANB_ind_ = (0.42 × (SNA) + 0.31 × (SN-MP) – 41.1) for males; ANB_ind_ = (0.31 × (SNA) + 0.20 × (SN-MP) – 28.9) for females [17].

Very recently, Paddenberg et al. [18] established improved and extended regression equations for equations derived by Panagiotidis/Witt and Järvinen for the individualized ANB and Wits appraisal [18].

It is well documented that individualized ANB and Wits appraisal are considered to be more useful cephalometric parameters for diagnosing skeletal class, because they are based on individually determined norm values instead of empirical norms, representing a population’s mean value.

According to a cross-sectional study done on SCIIIMO South Korean and Spanish participants, the results of the varimax factorial analysis (VFA) and cluster analysis (CA), showed a distinct distribution of the two ethnic groups, as well as differences within the same ethnic group [19]. According to Dehesa-Santos et al., cluster 1 was predominantly Spanish, and clusters 2 and 3 were mainly South Korean, with opposite phenotypes of mandibular projection and craniofacial pattern [19]. In another study that compared the craniofacial characteristics of skeletal and dental SCIIMO traits from Indian and Vietnamese individuals, it was found that the ANB angle was significantly greater in males (+ 1.4 deg) and females (+ 1.9 deg) in South Indian individuals. In addition, this study detected differences in the plane angle, articular angle, anterior facial height, and lower anterior facial height and described that SCIIMO was more severe in South Indian compared to Vietnamese adults [20]. However, to our knowledge there is no study comparing the cephalometric parameters between different subgroups of Arab patients, presenting skeletal class II or III.

Over the last decade, artificial intelligence (AI), especially machine learning (ML), has emerged in the field of dentistry, and assists the clinicians in the processing of images as well as in treatment decisions [10]. In addition, deep learning algorithms has been applied in the cephalometric analysis, and many approaches have focused on the detection of cephalometric landmarks [21].

The non-uniformity among orthodontics regarding the definition of these landmarks, in addition to the quality of the image, leads to significantly different outcomes of landmark coordinates and geometrical parameters [10]. The most common AI fields in dentistry are classification, regression, detection, and segmentation [10]. The constraint with all the currently available equations for individualizing the ANB angle and Wits appraisal is the fact there are many different and various equations that were used.

Therefore, the primary aim of this study was to derive a new machine learning model, based on the most important parameters and results from principal component analysis, to correctly identify Palestinian Arab residents of Israel as skeletal class II or III. We intended to use various machine learning models and different input variables to detect the best fitting one. Furthermore, additional analysis were done to investigate the effect of age and gender specific subgroups. It was the null hypothesis of our study that machine-learning models without using the classic equations of Calculated_ANB, won’t be able to classify either its SCIIMO or SCIIIMO with a high accuracy.

First, we applied a general Machine Learning (ML) model that included all parameters to classify the patients. Then, after excluding the Calculated_ANB and measured ANB, the most important variables were used to conduct a stepwise machine-learning process. The machine-learning models that were performed to classify patients as SCIIMO or SCIIIMO are- **Linear Discriminant Analysis (LDA)**, **Support Vector Machine (SVM)**, **K-Nearest Neighbors (KNN**), **Random Forest (RF)**, and classification and regression trees (CART).

## Materials and methods

### Ethical statement

All human samples presented in this study were assessed and treated according to current guidelines and followed the regulations of the Ethics Committee of the University of Regensburg. The committee had reviewed and approved this research project and study design with approval number 19-1596-101 (dated 13.11.2019). All patients were assessed and treated at the Orthodontic Research Center based in Jatt, Israel, and agreed to participate in this quantitative, observational study after detailed explanation by signing a corresponding consent form. The experimental data consisted of the coded records of 502 patients, who were Palestinian Arab citizens of Israel and diagnosed as skeletal class II or III. All data were collected as part of the routine orthodontic diagnostics, which had been taken for the purpose of the orthodontic treatment only. The research sample consisted of 502 patients with skeletal class II (*n* = 237, 47.21%) and III (*n* = 265, 52.78%).

The sample size was determined by the maximum number of cases, presenting skeletal class II and III, available within the period of recruitment. In addition, each machine-learning model was cross-validated to estimate its performance on unseen data in correctly classifying, and the sample size was sufficient to get the desired accuracy results.

Only orthodontic patients assessed and treated at the Orthodontic Research Center based in Jatt, Israel, were included in this study.

The inclusion criteria were, 1.Patients diagnosed with skeletal class II (Calculated_ANB > 1) or skeletal class III (Calculated_ANB<-1) according to the definition of Panagiotidis and Witt (Calculated ANB = ANB – individualized ANB of Panagiotidis and Witt [14]); and 2. Patients with pre-treatment lateral cephalograms available.

### Cephalometric variables

The cephalometric parameters included in this study analysis, with complete information and location, are presented in Supplementary Fig. 1A and described in Supplementary Table 1. In the present study, patients were included according to the Calculated_ANB. In fewer cases, they were included and diagnosed SCIIMO and SCIIIMO, even when they were not in the expected range of the Calculated_ANB that was proposed by Panagiotidis and Witt, but according to the orthodontist’s team clinical diagnosis, and according to other crucial parameters, like ANB angle and Wits appraisal. The fact that the Calculated_ANB doesn’t fit all cases is expected and was examined by Panagiotidis and Witt and can be explained by the correlation coefficient of the ANB_ind_ equation, *r* = 0.808 [14].

The mean age of skeletal class II patients was 17 (M = 17, SD = 6.5), with an age range of 7–44. Among class II patients, females constituted more than half (*n* = 162,68%). Concerning skeletal class III patients, the mean age of the patients was 18 (M = 18, SD = 8.1), with an age range of 6–54, and here also, females were more than half of the patients in this class (*n* = 140,52.83%). Supplementary Tables 2A  and 2B summarize the full detailed information about the tested SCIIMO and SCIIIMO patients, respectively.

### Data analysis

Data analysis was performed using the R software platform using the one-way analysis of variance tests (ANOVA). Post-hoc analysis was used to understand the differences between different subgroups of gender and age within the same and other classifications.

In addition, to understand the correlations between the different cephalometric parameters among the various (sub)groups, the Spearman Correlation was applied and visualized as a Heatmap correlation matrix. Furthermore, to estimate better our data structure and to gain thorough knowledge about the most informative and variant cephalometric parameters in our data, Principal Component Analysis (PCA) was done to identify if the information of the cephalometric parameters is well represented by the principal components chosen. We used different figures to illustrate the importance, but also the weight for each cephalometric parameter when calculating the principal component. In this study, we analyzed the first four components that explained about 92% of the variation in our data. Finally, machine-learning models were applied to examine our main hypothesis.

### Machine learning methods

LDA was proposed by R. Fischer in 1936. It consists of finding the projection hyperplane that minimizes the interclass variance and maximizes the distance between the projected means of the classes [22]. The next model we used was **the SVM model**,** which** implements the following idea: input vectors are non-linearly mapped to a very high-dimension feature space. In this feature space, a linear decision surface is constructed [23]. This model can be relatively simple and flexible for addressing various classification problems. SVMs distinctively afford balanced predictive performance, even in studies where sample sizes may be limited [24]. We also applied the nearest neighbor decision (KNN) rule, which assigns to an unclassified sample point the classification of the closest of a set of previously classified points [25, 26]. This study used Accuracy to select the optimal model using the most considerable value. The final value used for the model that includes Wits appraisal only was k = 9 (9 neighbors), and k = 7 (7 neighbors) for the model that contains Wits appraisal and SNB angle. In addition, we applied **RF model**, which uses many decision trees. This algorithm is a combination of tree predictors such that each tree depends on the values of a random vector sampled independently and with the same distribution for all trees in the forest [27, 28]. Finally, we applied the CART model, and the data was partitioned along the predictor axes into subsets with homogeneous values of the dependent variable, a process represented by a decision tree that can be used to make predictions from new observations [29].

### Classification models

Classification models - Linear Discriminant Analysis (LDA), Support Vector Machine (SVM), K Nearest Neighbor (KNN), Random Forest (RF), and Classification and Regression Tree (CART). They were all applied using the K-fold cross-validation (K = 10) implementation of the R package Caret.

### Model validation

We validated our models using the k-fold cross-validation approach. Cross-validation provides a simple and effective method for both model selection and performance evaluation; under k-fold cross-validation, the data are randomly partitioned to form k-disjoint subsets of approximately equal size [30, 31]. K (10)-fold cross-validation was employed in this research. Finally, we used a separate validation set (30%) to provide a more reliable estimate of model performance on unseen data and visualized the data on a confusion matrix.

## Results

### Comparison of cephalometric parameters

Our observations show that there are variations in cephalometric parameters in different gender and age specific subgroups within the same skeletal malocclusion class, and between the different classes. To evaluate the effect of the potential confounders gender and age on the cephalometric measurements, we compared each group with the other groups by conducting multiple comparison tests. Table 1A and 1C show the multiple tests performed, and the adjusted p-values were obtained by Tukey test. Values were regarded as significant at *p* < 0.01 and *p* < 0.05.

**Table 1A:** Multiple groups comparisons of cephalometric parameters using the Tukey method. Significant differences are indicated by p-values less than 0.01 and 0.05. Compares by gender within the same class

Parameter	Group A _ Group B	Difference	Lower CI	Upper CI	Adj. *P* value
Class II					
S-N (mm)	II_Male-II_Female	2.35	0.49	4.22	0.01
**Class III**					
NL-NSL angle	III_Male-III_Female	-1.22	-2.32	-0.11	0.02
PFH/AFH ratio	III_Male-III_Female	2.83	1.09	4.57	0.00
SNA angle	III_Male-III_Female	1.10	0.10	2.11	0.03
SNB angle	III_Male-III_Female	1.76	0.13	3.38	0.03
SN-Ba angle	III_Male-III_Female	-2.96	-4.71	-1.21	0.00
SN-Pg angle	III_Male-III_Female	2.17	0.78	3.56	0.00
ML-NSL angle	III_Male-III_Female	-3.31	-5.68	-0.93	0.00
+ 1/NL angle	III_Male-III_Female	1.91	0.13	3.69	0.04
+ 1/SNL angle	III_Male-III_Female	2.99	0.12	5.85	0.04
+ 1/NA angle	III_Male-III_Female	2.23	0.43	4.03	0.02

### Comparison of cephalometric parameters within the same skeletal class group

The results of our analyses showed that the results of skeletal class III males presented a significantly more prognathic mandible (SNB) and anterior position of the chin (SN-Pg) than females. In the vertical direction and compared to females, skeletal class III males had a more horizontal growth pattern (PFH/ AFH) and a bigger counterclockwise rotation of the maxilla (NL-NSL) and the mandible (ML-NSL) (*p* < 0.05) (Table 1A).

Furthermore, the analysis of skeletal class II patients revealed that adult patients (age > 21 years) presented a significant more open vertical configuration than younger ones, as evident from the divergence of the jaw bases (NL-ML) and the inclination of the mandible (ML-NSL) (*p* < 0.01). In line with this, the growth pattern was more vertical in adults compared to younger individuals, according to the parameters PFH/ AFH and facial axis (*p* < 0.05). As shown by the Gonion angle, adolescents (age 14–20) showed a more horizontal growth pattern compared to children (age 0–13), as well as more retroinclined and retropositioned upper front teeth (*p* < 0.05) (Table 1B).

**Table 1B:** Compares by age within the same class

Parameter	Group A _ Group B	Difference	Lower CI	Upper CI	Adj. *P* value
Class II					
NL-ML angle	II_Age > 21-II_14 < Age < 20	4.21	1.06	7.36	0.00
PFH/AFH ratio	II_Age > 21-II_14 < Age < 20	-2.66	-5.28	-0.05	0.04
PFH/AFH ratio	II_14 < Age < 20-II_0 < Age < 13	1.98	0.11	3.85	0.03
Gonial angle	II_14 < Age < 20-II_0 < Age < 13	-3.01	-5.68	-0.34	0.02
Gonial angle	II_Age > 21-II_14 < Age < 20	3.37	0.46	6.28	0.02
Facial axis	II_Age > 21-II_0 < Age < 13	-2.50	-4.39	-0.61	0.01
Facial axis	II_Age > 21-II_14 < Age < 20	-2.42	-4.18	-0.66	0.00
SNB angle	II_14 < Age < 20-II_0 < Age < 13	2.00	0.08	3.91	0.04
SN-Pg angle	II_14 < Age < 20-II_0 < Age < 13	1.31	0.09	2.52	0.03
SN-Pg angle	II_Age > 21-II_14 < Age < 20	-1.60	-2.93	-0.28	0.01
ML-NSL angle	II_Age > 21-II_14 < Age < 20	3.99	0.45	7.54	0.02
+ 1/NA angle	II_14 < Age < 20-II_0 < Age < 13	-5.29	-9.54	-1.03	0.01
+ 1/NA (mm)	II_14 < Age < 20-II_0 < Age < 13	-1.27	-2.29	-0.25	0.01
+ 1/NA (mm)	II_Age > 21-II_0 < Age < 13	-1.01	-1.96	-0.06	0.03
**Class III**					
Go-Me (mm)	III_14 < Age < 20-III_0 < Age < 13	4.15	1.00	7.30	0.00
Go-Me (mm)	III_Age > 21-III_0 < Age < 13	4.33	0.88	7.77	0.00
Wits appraisal	III_14 < Age < 20-III_0 < Age < 13	-2.04	-3.60	-0.48	0.00
Wits appraisal	III_Age > 21-III_0 < Age < 13	-2.56	-4.27	-0.85	0.00
ANBind	III_Age > 21-III_0 < Age < 13	0.66	0.06	1.27	0.03
Calculated_ANB	III_14 < Age < 20-III_0 < Age < 13	-0.87	-1.68	-0.06	0.03
Calculated_ANB	III_Age > 21-III_0 < Age < 13	-1.23	-2.12	-0.35	0.00
+ 1/NL angle	III_14 < Age < 20-III_0 < Age < 13	3.53	0.35	6.71	0.02
+ 1/NL angle	III_Age > 21-III_0 < Age < 13	4.28	0.80	7.75	0.01
+ 1/SNL angle	III_14 < Age < 20-III_0 < Age < 13	4.59	0.17	9.00	0.04
+ 1/SNL angle	III_Age > 21-III_0 < Age < 13	4.74	0.18	9.31	0.04
+ 1/NA angle	III_14 < Age < 20-III_0 < Age < 13	2.95	0.35	5.56	0.02
+ 1/NA angle	III_Age > 21-III_0 < Age < 13	4.00	1.14	6.86	0.00
+ 1/NA (mm)	III_14 < Age < 20-III_0 < Age < 13	1.13	0.11	2.15	0.02
+ 1/NA (mm)	III_Age > 21-III_0 < Age < 13	1.59	0.48	2.71	0.00
-1/NB angle	III_Age > 21-III_0 < Age < 13	2.81	0.06	5.56	0.04
-1/NB (mm)	III_Age > 21-III_0 < Age < 13	1.61	0.44	2.78	0.00
-1/NB (mm)	III_Age > 21-III_14 < Age < 20	1.18	0.14	2.23	0.02
Interincisal angle	III_Age > 21-III_0 < Age < 13	-6.07	-11.28	-0.87	0.01

Concerning the effect of age, among skeletal class III patients’ adults presented a more severe degree of the sagittal skeletal discrepancy (Wits appraisal) than younger ones (*p* < 0.05). Furthermore, the upper incisors of adults were more retroinclined (+ 1/NL, + 1/NSL) and anteriorly positioned (+ 1/NA (mm)) than in younger patients, although the interincisal angle was smaller in adults compared to adolescents and children (*p* < 0.05). Patients, aged 14–20 years, also had a more pronounced skeletal class III (Wits appraisal) (*p* < 0.05) and more retroinclined (+ 1/NL, + 1/NSL) and anteriorly positioned (+ 1/NA (mm)) upper incisors compared to children (*p* < 0.01) (Table 1B).

Moreover, adult females with skeletal class II presented more hyperdivergent jaw bases (NL-ML), and more posteriorly rotated mandible (ML-NSL) (*p* < 0.05). In line with the above-mentioned findings, according to the facial axis, these adult females with skeletal class II had a more vertical growth pattern than adolescent females (*p* < 0.01). Among males, the upper incisors were more retroinclined (+ 1/NA) in adolescents than in children (*p* < 0.05), but among females with skeletal class II, adolescents had more retroinclined lower incisors (1/ML) than children (*p* < 0.01). Regarding patients with skeletal class III, adult males presented a more pronounced sagittal skeletal discrepancy (Wits appraisal) than children. Furthermore, in male adolescents with skeletal class III the growth pattern (PFH/ AFH) was more horizontal than in female adolescents (*p* < 0.05) (Table 1C).

**Table 1C:** By both gender and age within the same class

Parameter	Group A _ Group B	Difference	Lower CI	Upper CI	Adj. *P* value
Class II					
NL-ML angle	II_Female_Age > 21-II_Female_14 < Age < 20	4.43	0.25	8.61	0.03
PFH/AFH ratio	II_Female_14 < Age < 20-II_Female_0 < Age < 13	3.08	0.23	5.93	0.03
PFH/AFH ratio	II_Female_Age > 21-II_Female_14 < Age < 20	-2.95	-5.81	-0.08	0.04
Facial axis	II_Female_Age > 21-II_Female_14 < Age < 20	-3.00	-5.49	-0.50	0.01
ML-NSL angle	II_Female_Age > 21-II_Female_14 < Age < 20	4.38	0.47	8.29	0.02
+ 1/NA angle	II_Male_14 < Age < 20-II_Male_0 < Age < 13	-9.27	-17.99	-0.55	0.03
-1/ML	II_Female_14 < Age < 20-II_Female_0 < Age < 13	5.34	0.06	10.61	0.04
**Class III**					
PFH/AFH ratio	III_Male_14 < Age < 20-III_Female_14 < Age < 20	3.79	0.45	7.14	0.01
SN-Ba angle	III_Male_14 < Age < 20-III_Female_14 < Age < 20	-4.29	-7.69	-0.90	0.00
S-N (mm)	III_Male_14 < Age < 20-III_Male_0 < Age < 13	4.55	0.18	8.92	0.04
Go-Me (mm)	III_Male_14 < Age < 20-III_Male_0 < Age < 13	6.02	0.67	11.38	0.01
Go-Me (mm)	III_Male_Age > 21-III_Male_0 < Age < 13	6.50	0.96	12.04	0.01
Wits appraisal	III_Male_Age > 21-III_Male_0 < Age < 13	-3.39	-6.14	-0.63	0.00
+ 1/SNL angle	III_Male_Age > 21-III_Male_0 < Age < 13	8.23	0.52	15.94	0.02
+ 1/NL angle	III_Male_Age > 21-III_Male_0 < Age < 13	5.24	0.49	10.00	0.02
-1/NB (mm)	III_Female_Age > 21-III_Female_0 < Age < 13	1.89	0.24	3.54	0.01
**Additional Variations**					
S-N (mm)	II_Male_0 < Age < 13-II_Female_14 < Age < 20	4.39	0.18	8.60	0.04
+ 1/NA angle	II_Male_14 < Age < 20-II_Female_0 < Age < 13	-9.97	-17.42	-2.52	0.00
PFH/AFH ratio	III_Male_14 < Age < 20-III_Female_Age > 21	4.13	0.18	8.07	0.03
SNB angle	III_Male_Age > 21-III_Female_0 < Age < 13	3.22	0.08	6.36	0.04
SN-Ba angle	III_Male_Age > 21-III_Female_14 < Age < 20	-4.15	-7.71	-0.60	0.01
SN-Pg angle	III_Male_Age > 21-III_Female_0 < Age < 13	3.59	0.36	6.81	0.02
SN-Pg angle	III_Male_Age > 21-III_Female_14 < Age < 20	2.87	0.06	5.68	0.04
Wits appraisal	III_Male_Age > 21-III_Female_0 < Age < 13	-3.14	-5.83	-0.44	0.01
Calculated_ANB	III_Male_Age > 21-III_Female_0 < Age < 13	-1.82	-3.21	-0.43	0.00
ML-NSL angle	III_Male_14 < Age < 20-III_Female_Age > 21	-5.12	-10.02	-0.21	0.04
+ 1/NL angle	III_Male_Age > 21-III_Female_0 < Age < 13	6.65	1.20	12.09	0.00
+ 1/NL angle	III_Male_14 < Age < 20-III_Female_0 < Age < 13	4.76	0.27	9.25	0.03
+ 1/SNL angle	III_Male_Age > 21-III_Female_0 < Age < 13	7.89	0.35	15.43	0.03
+ 1/NA angle	III_Male_Age > 21-III_Female_0 < Age < 13	6.75	2.03	11.47	0.00
+ 1/NA (mm)	III_Male_14 < Age < 20-III_Female_0 < Age < 13	1.61	0.09	3.13	0.03
+ 1/NA (mm)	III_Male_Age > 21-III_Female_0 < Age < 13	2.22	0.47	3.97	0.00
-1/NB (mm)	III_Male_0 < Age < 13-III_Female_Age > 21	-1.81	-3.49	-0.12	0.03
-1/NB (mm)	III_Male_14 < Age < 20-III_Female_Age > 21	-1.58	-3.13	-0.03	0.04
Interincisal angle	III_Male_Age > 21-III_Female_0 < Age < 13	-7.99	-15.05	-0.93	0.02

### Variation of cephalometric parameters between patients with different skeletal classes

Our results demonstrated a large variety of significant differences when comparing different skeletal classes and subgroups of gender and age, among which the most important parameters were Gonion angle, SNB angle, ANB angle, Calculated_ANB, SN-Pg angle, and Wits appraisal (Supplementary Table 3).

### Heatmaps of spearman correlation

#### Global heatmap correlation matrices of assessed cephalometric parameters under different classifications and sub-groups

**Fig. 1:**
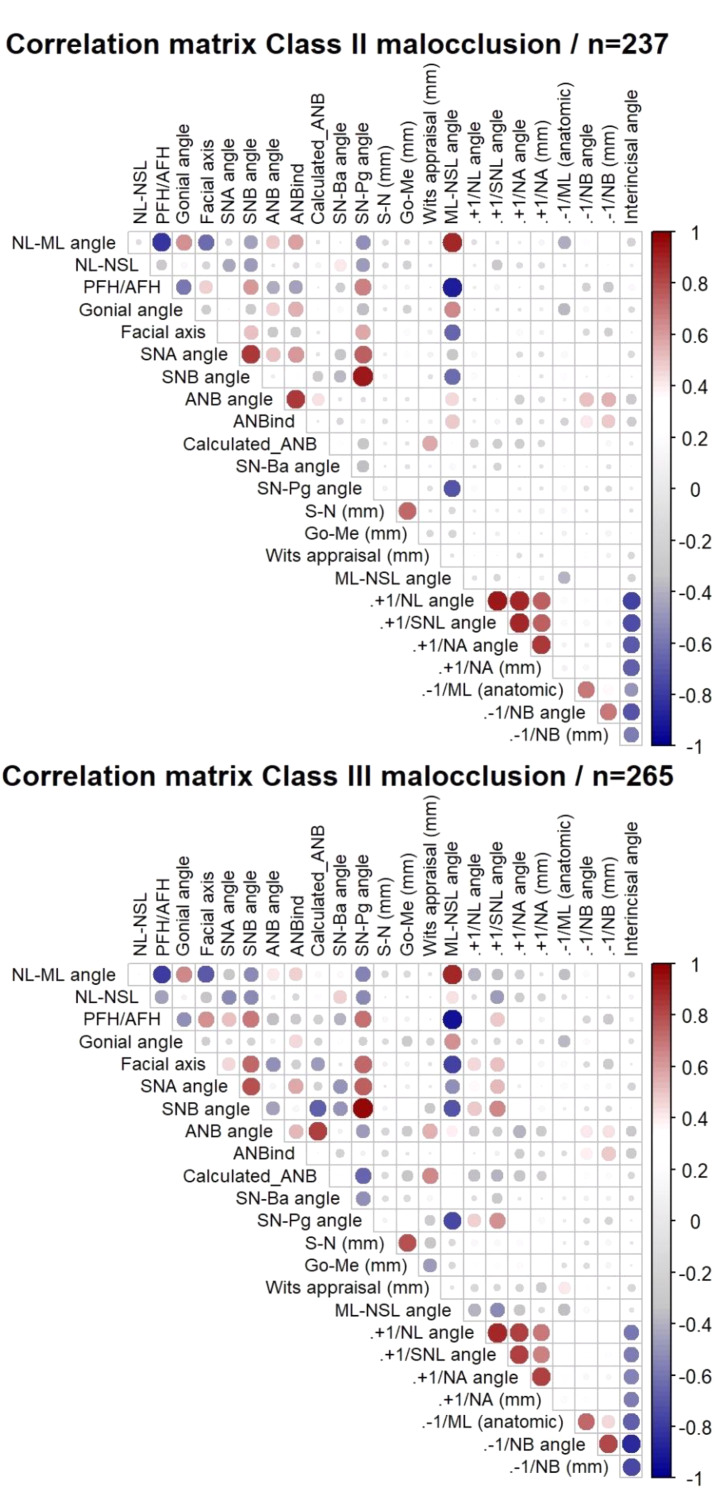
The heatmaps present the Spearman correlation between different cephalometric parameters for SCIIMO and SCIIIMO patients. Color coding signifies the strength and direction of the correlation: blue indicates a negative correlation (strongest at ρ = -1), red indicates a positive correlation (strongest at ρ = 1), and the intensity of the color reflects the correlation strength. This Figure shows SCIIMO and SCIIIMO correlations regardless of gender and age

The overall heatmap correlation matrices of cephalometric parameters in skeletal class II and III groups demonstrated many correlations between the variables. Results show a strong and significant correlation between parameters in the same dimension. In both skeletal classes, the results revealed many correlations between Calculated_ANB and other parameters. For example, among skeletal class II patients, Calculated_ANB presented significant correlations with the following skeletal variables: SNB (ρ=-0.274, *P* < 0.01), ANB (ρ = 0.430, *P* < 0.01), SN-Pg (ρ=-0.302, *P* < 0.01) and Wits appraisal (ρ = 0.574, *P* < 0.01). Regarding skeletal class III, Calculated_ANB was associated with the following skeletal parameters: Facial axis (ρ=-0.474, *P* < 0.01), SNB (ρ=-0.670, *P* < 0.01), ANB (ρ = 0.822, *P* < 0.01) SN-Pg (ρ=-0.644, *P* < 0.01), and Wits appraisal (ρ = 0.655, *P* < 0.01) (Fig. 1).

#### Gender and age variation

The heatmaps for each subgroup revealed many specific significant correlations, although they varied between different subgroups. Detailed results are available in the supplementary Tables 2A and 2B.

#### Principal component analysis (PCA)

We ran a PCA analysis with all cephalometric parameters to better estimate our data structure and gain thorough knowledge about the most informative parameters in our data. After normalizing our data, the results demonstrated that the first component explains more than half of the total variance (53%) and adding three further principal components lead to a cumulative proportion of variance of 92% (Table 2A). To better understand, which components are included in the first component, the loading matrix was calculated, showing high positive values for ANB, and Calculated_ANB, and ML-NSL. Furthermore, high negative values were identified for SN-Pg, SNB, and facial axis. Variables, which had a high impact on the second component were Gonion angle, ML-NSL, and NL-ML (positive), and − 1/ML, Wits appraisal, Calculated_ANB, and PFH/AFH ratio (negative). The specific details of all parameters are represented in Table 2B.

**Table 2A:** Results of a principal component analysis (PCA) performed on the cephalometric variables. Shows four principal component analyses (PCA 1–4) of the cephalometric variables. Columns of Comp.1, Comp.2, Comp.3, and Comp.4 show component 1, 2, 3, and 4 analyses, respectively, with the standard deviation for every component, the proportion of variance that each component explains, and the cumulative proportion of variance. The first four components explain 90% of the variance

	Comp.1	Comp.2	Comp.3	Comp.4
Standard deviation	1.34	0.79	0.73	0.42
Proportion of Variance	0.53	0.18	0.15	0.05
Cumulative Proportion	0.53	0.71	0.87	0.92

**Table 2B:** Presents the PCA loading matrix. Each cell reflects the contribution of a specific cephalometric parameter to a particular component (comp. 1–4). Positive values indicate a positive association, while negative values (shown in bold) indicate a negative association between the variable and the component

Parameter	Comp.1	Comp.2	Comp.3	Comp.4
NL-ML angle	0.22	**0.37**	0.09	0.10
NL-NSL	0.14	0.04	-0.08	**-0.35**
PFH/AFH	**-0.24**	**-0.33**	-0.03	0.14
Gonial angle	0.05	**0.40**	0.03	0.11
Facial axis	**-0.29**	-0.13	-0.02	-0.02
SNA angle	0.01	-0.04	0.01	0.16
SNB angle	**-0.30**	0.06	0.01	**0.26**
ANB angle	**0.31**	-0.21	0.06	0.14
ANB_ind_	0.15	0.17	0.13	**0.42**
Calculated_ANB (ANB – ANB_ind_)	**0.28**	**-0.31**	0.02	-0.01
SN-Ba angle	0.16	-0.02	-0.04	-0.37
SN-Pg angle	**-0.34**	0.02	0.01	**0.24**
S-N (mm)	-0.04	-0.07	0.04	**-0.34**
Go-Me (mm)	-0.15	0.02	0.02	-0.29
Wits appraisal (mm)	**0.24**	**-0.33**	0.03	0.08
ML-NSL angle	**0.26**	**0.36**	0.04	-0.08
+ 1/NL angle	-0.19	0.01	**0.36**	-0.11
+ 1/SNL angle	-0.20	-0.02	**0.32**	0.07
+ 1/NA angle	-0.17	0.07	**0.32**	-0.19
+ 1/NA (mm)	-0.16	0.12	**0.34**	-0.24
-1/ML (anatomic)	0.11	**-0.36**	0.23	0.00
-1/NB angle	0.17	-0.08	**0.34**	0.14
-1/NB (mm)	0.15	0.05	**0.34**	0.04
Interincisal angle	-0.09	0.08	-0.47	0.01

**Fig. 2:**
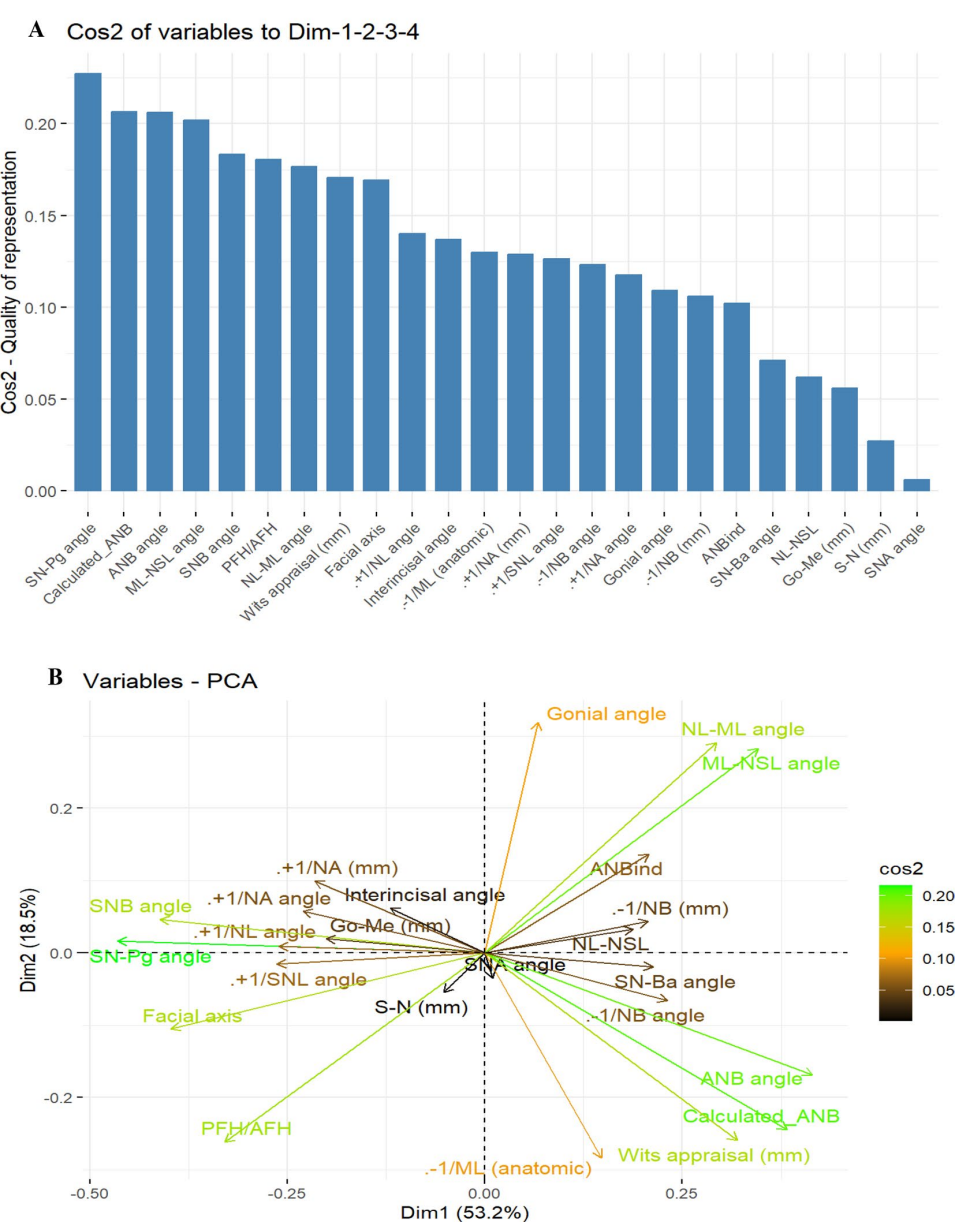
The results of a Principal Component Analysis (PCA) on the assessed cephalometric parameters. Figure 2**A** shows the contribution of each cephalometric parameter to the first four principal components (PCs) through their cosine squared values. The X-axis lists all variables, and the Y-axis shows the values of the Cos2 quality of the presentation. Variables with the highest contributions to the first four PCs are SN-ML angle, ML-NSL angle, SN-Pg angle, and PFH/AFH ratio. Figure 2**B** presents a PCA biplot that visualizes the relationships between the variables and the first two PCs (PC1 and PC2). PC1 captures 51.2% and PC2 captures 19.3% of the data variance. The X and Y axes represent PC1 and PC2, respectively. High contributing variables (identified in Fig. 2A) are colored green, while variables with lower contributions are shown in black. This combined analysis helps to understand how the original variables relate to the first four components and identify the most influential factors contributing to the variation in the data

Subsequently, we calculated the contribution of each parameter to the first four components using the cosine squared function. The results showed that the parameters SN-Pg, Calculated_ANB, ANB angle, and ML-NSL contributed the most to the first four components (Fig. 2A). Finally, as presented in Fig. 2B, we observed a similar result with a different visualization.

### Machine learning models

**Fig. 3:**
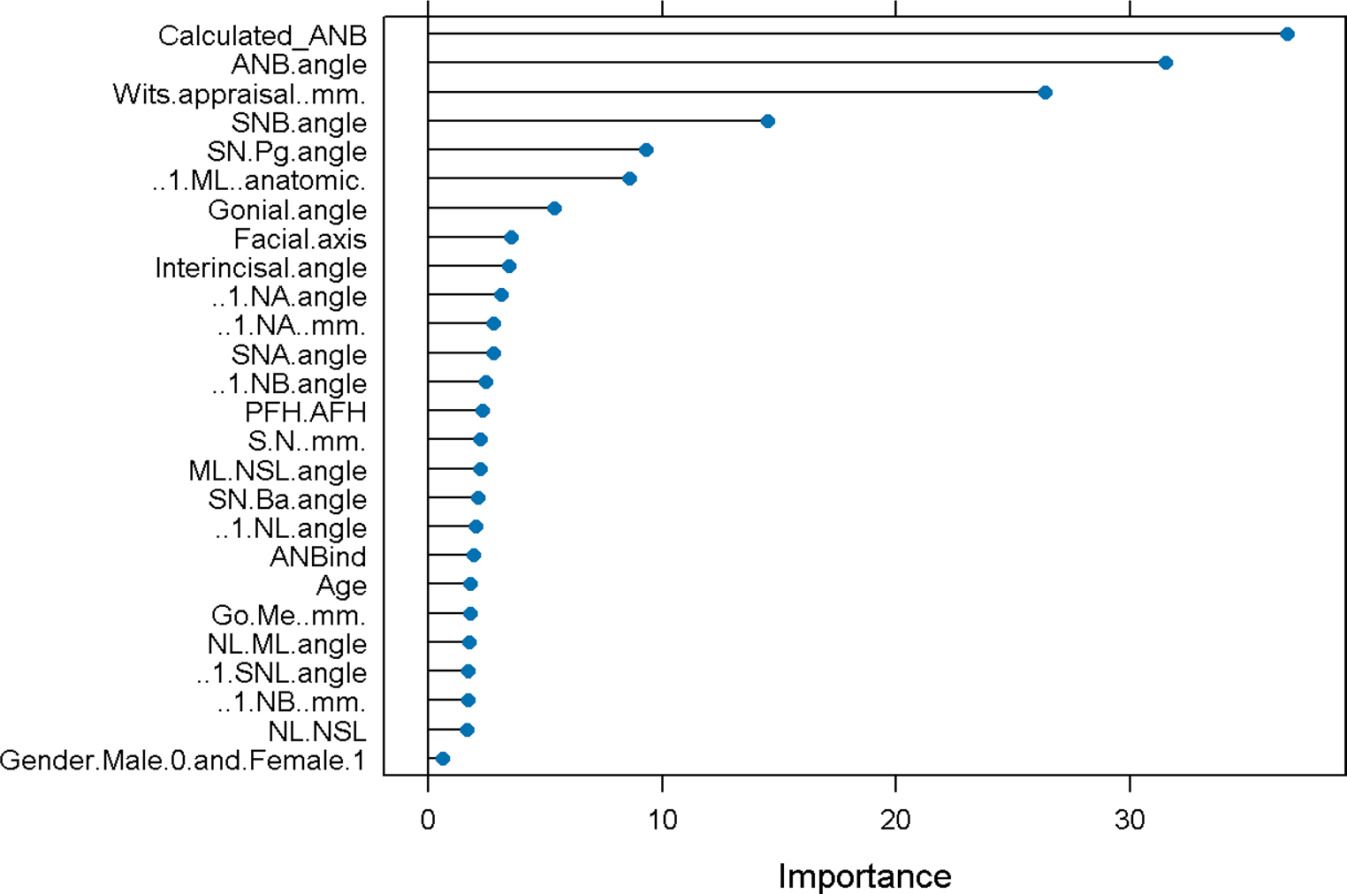
Summary of the General Machine Learning model showing the importance of each parameter to the model in predicting SCIIMO or SCIIIMO. X-axis shows the prediction importance of the different assessed variables. Y-axis shows the list of the assessed variables

The main aim of this study was to establish machine learning (ML) models to increase the precision in the diagnosis of skeletal class II and III instead of applying the individualized ANB of Panagiotidis and Witt, combined with the measured ANB only. When we tested different ML models based on all parameters (general model) in the LDA and RF models, we received 0.99 accuracy (Accuracy = 0.99, Kappa = 0.99) in the classification of skeletal class II and III. Then, we analyzed the performance of ML models, which varied according to the ML-type and the amount of input parameters. We used that general model that contained all the input variables to estimate the importance of each parameter to the model, thereby determining the other models to be evaluated (Fig. 3).

**Fig. 4:**
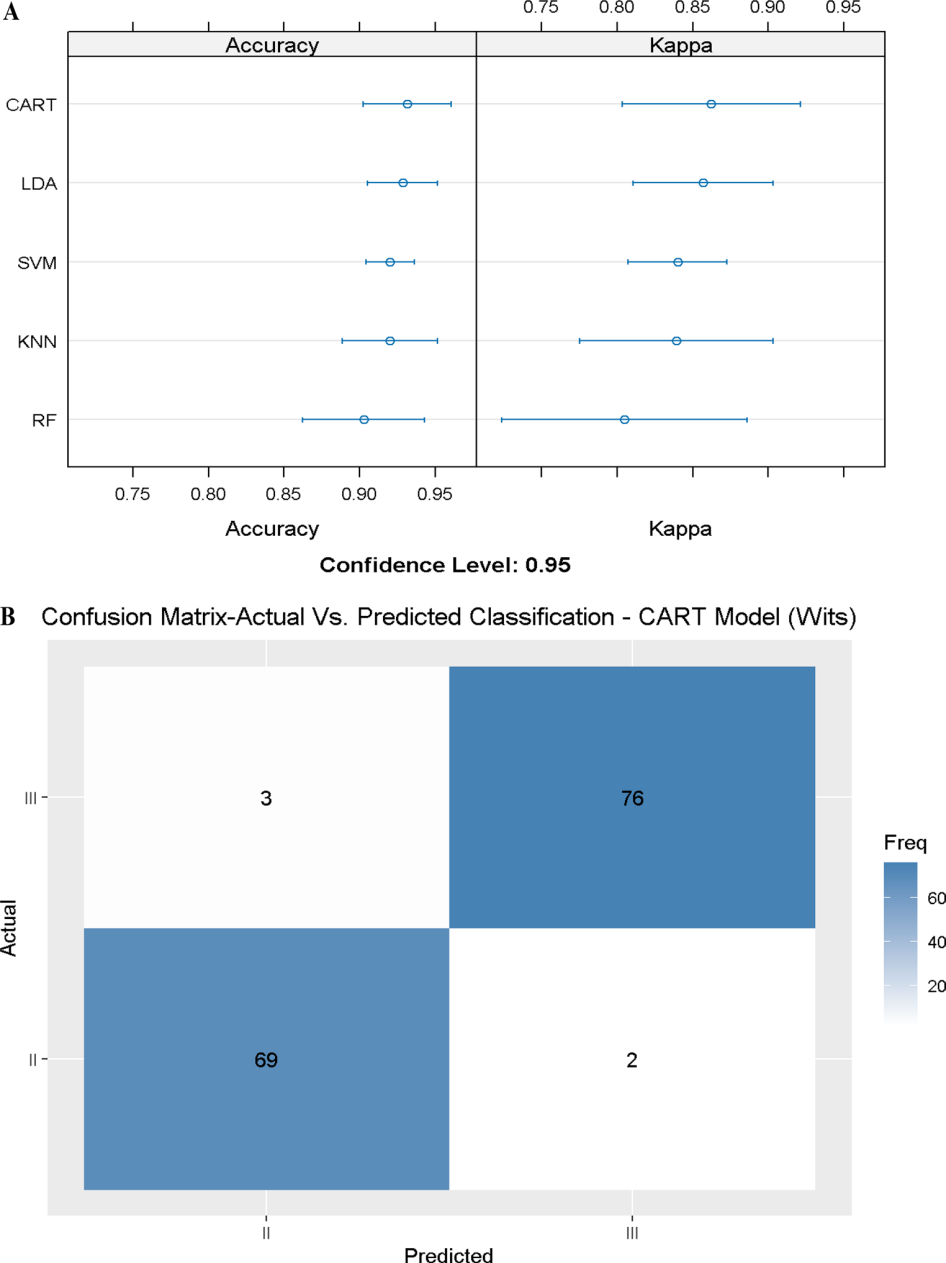
Summary of model 1 (one predictor) of the different Machine Learning models. Figure 4**A** presents a **S**ummary of the five Machine-Learning classification models, including Linear Discriminant Analysis (LDA), Support Vector Machine (SVM), K-Nearest Neighbors, Random Forest (RF), Classification, and Regression Tree (CART), which are presented on the Y-axis. The X-axis shows the Accuracy and Kappa scores for each model. The first model included the Wits appraisal only; in the LDA and SVM models, we received an Accuracy of 0.90 and Kappa of 0.80, while Fig. 4**B** presents the LDA Machine Learning Model Confusion Matrix for Wits appraisal to predict the classification (Predicted) compared to the Actual classification, based on using this variable only. The X-axis shows the SCIIMO and SCIIIMO predictions, and the Y-axis shows the number of identified patients in each classification

In the first stage, we tested an ML model using only the most important variable that followed the Calculated_ANB and measured ANB angle. Hence, the first model included the Wits appraisal only and achieved an accuracy of 0.93 (Accuracy = 0.93, Kappa = 0.86) in the CART model (Fig. 4A-B). The second model included the Wits appraisal and the SNB angle, increasing the accuracy to 0.95 in the SVM model. Finally, adding the third slightly improved the performance of the machine learning models (Table 3).

**Table 3:** Stepwise Forward Machine Learning Models, including General model, model 1, model 2, and model 3: these rows represent different models used for prediction, potentially containing various combinations of the cephalometric parameters. The general model included all parameters. In models 1–3, the sign (-) indicates that the parameter was not included, while (+) indicates that the parameter is included

	Wits appraisal (mm)	SNB angle	SN-Pg angle	Best Model	Accuracy	Kappa
All parameters included - general model				RF	0.99	0.99
Model 1	(+)	(-)	(-)	CART	0.93	0.86
Model 2	(+)	(+)	(-)	SVM	0.95	0.91
Model 3	(+)	(+)	(+)	KNN	0.968	0.93

**Fig. 5:**
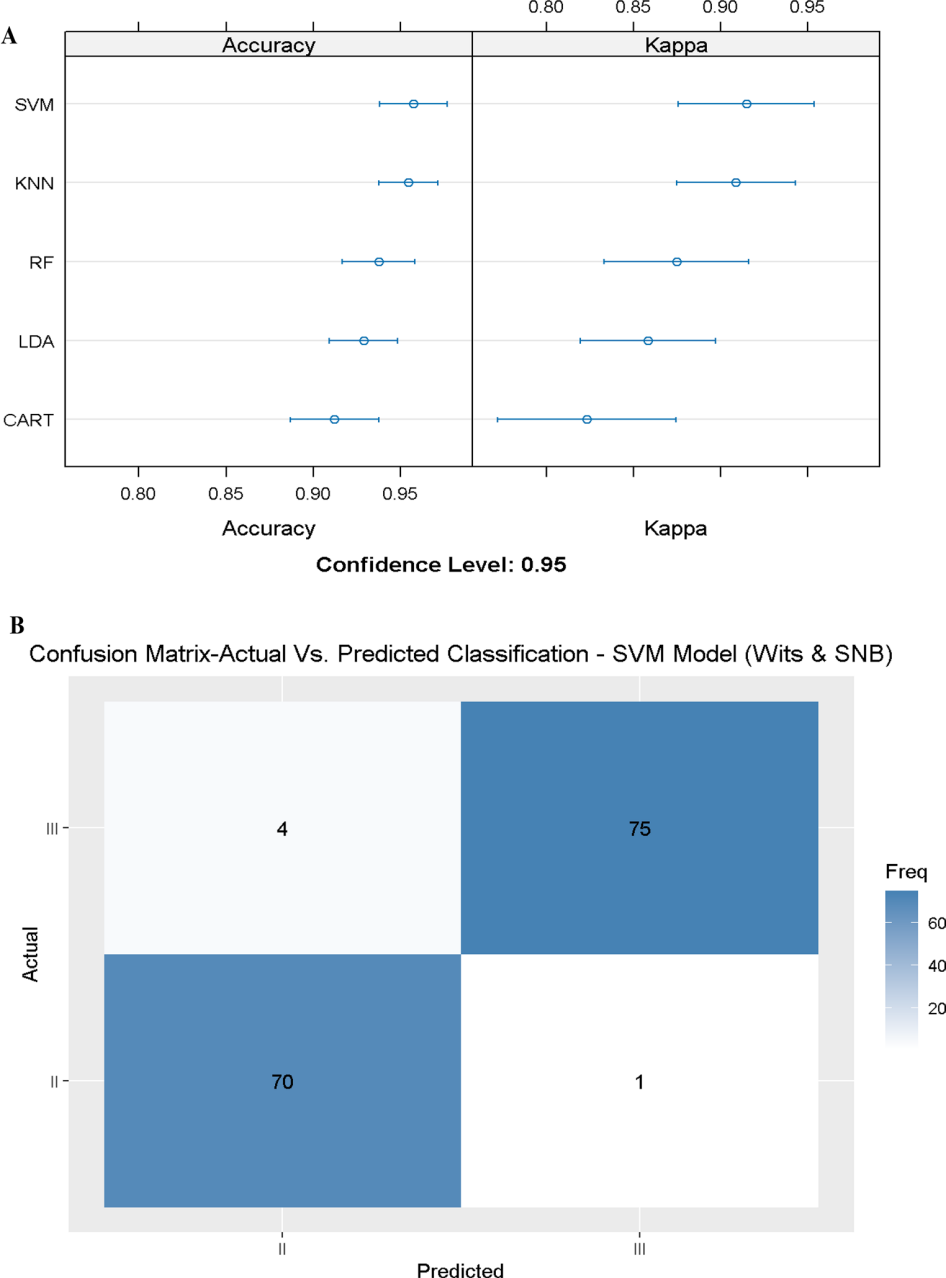
Summary of model 2 (two predictors) of the different Machine Learning models. Figure 5**A** presents a summary of the five Machine-Learning classification models tested, including Linear Discriminant Analysis (LDA), Support Vector Machine (SVM), K-Nearest Neighbors, Random Forest (RF), Classification and Regression Tree (CART) as presented on the Y-axis. The X-axis shows the Accuracy and Kappa scores for each model. At the same time, Fig. 5**B** presents the Machine Learning Model Confusion Matrix, which shows the ability of the KNN model to predict the classification (Predicted) compared to the Actual classification based on Wits appraisal and SNB angle. The X-axis shows the SCIIMO and SCIIIMO predictions, and the Y-axis shows the number of identified patients in each classification

The results of the machine learning models that include the first two variables (Wits appraisal and SNB angle), were satisfying in classifying patients as skeletal class II or III. The highest mean accuracy value was obtained by the models SVM, and KNN, with an accuracy of 0.95 (Kappa SVM = 0.91, Kappa KNN = 0.908). The model LDA,, and RF revealed a high accuracy of approximately 0.93 too (Accuracy = 0.93, Kappa LDA = 0.85, Kappa RF = 0.87). Finally, the CART model also had a high accuracy score of 0.91 (Accuracy = 0.91, Kappa = 0.82) (Fig. 5A).

Finally, 30% of the recruited patients were used to validate the ML model by comparing the classification obtained by the ML model with the classification made by Calculated_ANB.70 skeletal class II patients were classified as class II both by the model and by the Calculated_ANB, 75 skeletal class III patients were classified as class III both by the model and by the Calculated_ANB. (Fig. 5B). To understand the confounding effect of gender and age, we repeated the previous model with same cephalometric parameters. We included gender and age as additional variables, and from the results, we can understand that the addition of gender and age did not improve the accuracy.

### Discussion

This research’s main objective was to establish a machine learning model that accurately determines the skeletal class II or III of Arab individuals. Secondary aims included comparing cephalometric parameters and their correlations between skeletal class II and III (sub)groups. We intended to illustrate the association of vertical and sagittal cephalometric parameters to define the sagittal discrepancy of the jaw bases with high precision, improving individualized diagnostics and treatment planning in orthodontics. For this purpose, first, cephalometric parameters were compared between skeletal class II and III patients and between age and gender-specific subgroups. Then, correlations between all cephalometric variables, including Calculated_ANB, were assessed for all skeletal class II/ III patients and the different subgroups concerning age and gender. Next, following PCA to detect the most relevant parameters in skeletal class II/ III diagnosis, we finally evaluated the primary outcome, i.e., the performance of machine learning models. During this process, different ML models in terms of the kind of model and the amount of input variables were tested to identify the best fitting one. We used the Calculated_ANB, i.e., the difference between the individualized ANB of Panagiotidis and Witt and the measured ANB, as a reference test to validate the ML model and to determine its accuracy, reliability (kappa), sensitivity, and specificity. Finally, we could reject the null hypothesis, as we established an accurate machine learning model for diagnosing skeletal class II/ III and identified significant differences in cephalometric measurements between different skeletal malocclusion and age and gender-specific subgroups.

### Different groups comparisons

The results showed that that there were many significant differences between gender and age specific subgroups. Although we identified some differences in cephalometric variables between subgroups of the same skeletal class, most significant differences were detected between subgroups of different skeletal classes. Many previous studies did not find that there is no significant difference between males and females [32]. For example, In research that studied the Lateral cephalograms of 105 Chinese subjects with Class II, there were no significant differences were detected between males and females for any of the parameters between males and females [32]. These results were also supported by Sharma and Xin [38],

who identified only small gender specific differences for six parameters. However, another study reported significant differences in cephalometric parameters between male and female adolescents, although variations were found for different malocclusion classes. In class II males, the maxilla was placed more protrusively (R2ANS; R2A) and the mandible was found to be larger both in the position and dimension (CoGn; R2M), whereas in class III adolescents no significant differences were detected [33]. Contrary to these findings, we did not observe any significant variation between male and female skeletal class II patients.

#### According to our results, age was identified to influence cephalometric measurements significantly

In summary, older patients revealed more hyperdivergent jaw bases (NL-ML), posteriorly rotated mandibles (ML-NSL), retroinclined (+ 1/NA angle) and retropositioned (+ 1/NA (mm)) upper incisors. According to van Diepenbeek et al. study, which investigated age-dependent changes of the parameters SNA, SNB, ANB and SN/GoMe in white adolescents, who were aged between 9 and 14, partly different results were described: the degree of prognathism of the maxilla (SNA: 0.1–0.3 per year) and the mandible (SNB: 0.2–0.4 per year) increased, although the sagittal discrepancy between the jaw bases (ANB: 0.1–0.2 per year) decreased at higher ages. Furthermore, van Diepenbeek et al. described a counterclockwise rotation of the mandible (SN/GoMe: 0.2–0.6 per year) [34]. In contrast, our results revealed a clockwise rotation of the mandible (ML-NSL) at higher ages, which might be explained by differences in the study populations.

Considering cephalometric parameters of skeletal class III patients, our findings presented several significant differences with respect to gender and age. In male subjects, both jaw bases were more anteriorly inclined (NL-NSL, ML-NSL), the growth pattern was more horizontal (PFH/ AFH), the mandible (SNB) was more prognathic and the sagittal position of chin (SN-Pg) was more anterior compared to females. Contrary to our results, Taner et al. [33] found mostly similar sagittal and vertical skeletal cephalometric variables in male and female skeletal class III children, aged between 10 and 11.5 years, except for the posterior (R_2_PNS) and anterior (R_2_ANT) nasal spine position, being more anterior in males. This contradicting finding might be due to differences in the study population and the method used to determine skeletal class (ANB vs. Calculated_ANB). Regarding the effect of age on cephalometric parameters in our skeletal class III sample, adult patients (age > 21) demonstrated a more pronounced skeletal class III (Wits appraisal) than younger subjects. Furthermore, skeletal class III adults presented more retroinclined (+ 1/NL angle, + 1/NSL angle) and anteriorly positioned (+ 1/NA (mm)) upper incisors, but a smaller interincisal angle compared to younger patients. Similarly, among skeletal class III patients, adolescents (age 14–20) showed a more severe skeletal class III (Wits appraisal) as well as more retroinlcined (+ 1/NL angle, + 1/NSL angle) but anteriorly positioned (+ 1/NA (mm)) upper front teeth compared to children (age 0–13). These findings demonstrate that the skeletal sagittal discrepancy increases during growth, and that upper incisors partly (sagittal position only) compensate the skeletal disharmony. Furthermore, this skeletal observation can be supported by the results of van Diepenbeek et al. [34], who reported a reduction in ANB-angle and, hence, a trend towards a more mesial basal relation during growth.

Regarding the correlations between cephalometric parameters, we detected many between patients with skeletal class II and III, especially between variables measured in the same dimension. Furthermore, many correlations were found between Calculated_ANB and other parameters in both skeletal classes. These findings demonstrate that skeletal class, determined by Calculated_ANB, mainly depends on other sagittal skeletal parameters and some vertical variables. Our findings are supported by several other studies already published. Using bivariate analysis, Jan et al. [35] showed that the ANB angle and Wits appraisal were significantly correlated (ρ = 0.469, *P* = 0.00). Moreover, another study identified statistically significant correlations between seven sagittal parameters. The correlation was robust between AXB and AF-BF distance, A-B plane and ANB angle, AXB and FABA, and AF-BF distance and FABA, but weak between ANB and beta angle [36]. Saad et al. [37] reported the most statistically significant and robust correlation between Calculated_ANB (with a different formula) and ANB, followed by the Wits appraisal. In addition, this study found that Calculated_ANB was not significantly associated with SNA and SN-GoMe.

The results of the PCA, which considered all cephalometric parameters, were very satisfying, as PC1 explained more than half of the variance, and the addition of PC2 + PC3 + PC4 resulted in a cumulative proportion of variance of 92% in the cephalometric data generated by skeletal class II/ III.

Among all cephalometric variables, the most important ones contributing to the first PC were the anteroposterior relationship (ANB angle, and Calculated_ANB, positive), the inclination of the mandible (ML-NSL, positive) as well as the sagittal position of the chin (SN-Pg) and mandible (SNB), the facial axis and PFH/ AFH (negative). Concerning the second principal component, the most relevant parameters according to their loading values were the growth pattern (Gonion angle), inclination of the mandible (ML-NSL), divergence of the jaw bases (NL-ML) (positive) as well as the skeletal class (Wits appraisal, Calculated_ANB, ANB) and inclination of the lower incisors (-1/ ML) (negative). These results demonstrate that both sagittal and vertical skeletal parameters influence the true anteroposterior discrepancy between the upper and the lower jaw.

In a previous study that performed PCA with cephalometric results found that 68.2% of the total sample’s shape variability was explained by the first 5 principal components. The most important parameter of the first principal component, which explained 29% of the variability, was the divergence of skeletal pattern, and the most relevant variable contributing to the second principal component, which added 20% to the total variance, was the anteroposterior maxillary relationship [38]. Another study, which evaluated 16 measurements using Steiner analysis [12] for 120 patients, identified five principal components, which covered 88.545% of the total variance of variables. The rotated components matrix showed that the PCs corresponded to the following measurement order: SND, Maxl-NA, 1I-NB S-E, and ANB [39].

The Machine Learning model, which included the Wits appraisal and SNB only, revealed an accuracy of 0.95, suggesting that the application of two cephalometric parameters only may be sufficient to diagnose an Arab individual as skeletal II or III because considering all variables in the general models added only 4% in accuracy. This finding demonstrates the necessity of accurately identifying the corresponding landmarks of the Wits appraisal and SNB. Even though the number of included parameters (Wits appraisal, SNB) in the ML model is comparable with the one used in the method suggested by Panagiotidis and Witt (SNA, ML-NSL, ANB), an advantage of our technique is its possible application in potential future feasibility studies, allowing an automated diagnosis of skeletal class II and III. Furthermore, future studies might combine the results of this study with a computerized detection of landmarks in lateral cephalograms, which might help orthodontists in precise and fast diagnosis of an individual’s skeletal class. Still, bigger sample sizes and validations in new data sets are necessary to develop such systems for clinical applications.

Another application of ML models in the field of orthodontics was described by Taraji et al. [40]. They analyzed lateral cephalograms and dental records of 182 post circumpubertal participants with skeletal and dental class III and aimed to identify critical morphological features that influence the decision camouflage vs. surgery in treatment planning to develop a machine learning model. According to their findings neither gender nor age were significantly different between groups, whereas Wits appraisal, anterior overjet and Mx/Md ratio were found as a key predictors [40].

This study presented a high accuracy in the ML model in Arab patients, but it’s important to check if similar results will be gained from other ethnic groups. For this purpose, in another study that it’s still under review, we examined SCIIMO and SCIIIMO German patients ML models, and found that Wits appraisal as an input variable only resulted in accuracy of 94.9% in the RF model (study under review). In another study that was done on all skeletal classes orthodontic patients in Sri Lanka, using cephalometric radiographs, and categorized patients according to the measured ANB, found that the accuracy of the multinomial logistic regression model, k-NN algorithm, random forest, and Naïve Bayes classification of malocclusion patterns are 88.89%, 83.33%, 88.89%, and 55.56%, respectively, using SNA, SNB and ANB parameters [41].

### Limitations

The study pool comprised patients presenting only skeletal class II and III, whereas patients with skeletal class I were not included in this investigation. Hence, within this analysis, we could not prove the machine learning model’s performance, i.e., the sensitivity, specificity, reliability, and accuracy, in a broader population with all skeletal classes. However, this is of significant importance in orthodontic diagnostics. However, this study tried to cover several research questions with a specific focus on skeletal malocclusions, and therefore, future studies are intended to address this shortcoming. Another limitation was the partly heterogeneous size of the age- and gender-specific subgroups, which the retrospective allocation of patients can be explained into different (sub)groups. Again, future studies should try to ensure homogenous sample sizes.

### Conclusion and future research

Based on the results of our study, we emphasize that gender and age influence the cephalometric measurements in patients with skeletal class II and III. In addition, PCA can be an effective tool to simplify many cephalometric parameters to two PCs that explain 71% of the variability of skeletal class II/ III diagnosis. Finally, A machine learning model, which considered the Wits appraisal and SNB only, achieved a high accuracy of 0.95 in diagnosing skeletal class II/ III. In summary, the study provides valuable information about the complexity of cephalometric measurement in an Arab population and, more importantly, presents an accurate machine learning model for the identification of individuals as skeletal class II or III, which might support clinicians in fast and precise diagnosis, thereby advancing personalized orthodontic diagnostics and treatment planning.

## Electronic supplementary material

Below is the link to the electronic supplementary material.

Supplementary Material 1

## Electronic supplementary material

Below is the link to the electronic supplementary material.

Supplementary Material 2

## Data Availability

No datasets were generated or analysed during the current study.
