# Supplementary material for: Lateral cephalometric parameters among Arab skeletal classes II and III patients and applying machine learning models
Source: Clin Oral Investig. 2024 Sep 3;28(9):511. doi: 10.1007/s00784-024-05900-2 (PMC11369042; doi:10.1007/s00784-024-05900-2)
Supplement: Supplementary file 1 — Supplementary Material 1 [file 784_2024_5900_MOESM1_ESM.docx]

| **Dimension/Group** | **Parameter** | **Unit** | **Definition** |
| --- | --- | --- | --- |
| Vertical Analysis | NL/ML (anatomic) | ° | The angle between the NL and ML |
| Vertical Analysis | SNL/ML (anatomic) | ° | The inclination of the mandible (mandibular inclination) relative to the nasion-sella line (anterior skull base, SNL) |
| Vertical Analysis | NL/NSL | ° | The angle between Sella-Nasion-line (NSL = SN) and nasal line (Spa-Spp) |
| Vertical Analysis | PFH/AFH | % | The ratio between posterior (SGo) and anterior (NMe) facial height |
| Vertical Analysis | Gonial Angle | ° | The angle between ML and line GoAr at Gonion |
| Vertical Analysis | Facial axis | ° | The angle between the lines NBa and PtGN’ |
| Sagittal Analysis | Angle SNA | ° | The angle between Sella, Nasion, and point A |
| Sagittal Analysis | Angle SNB | ° | The angle between Sella, Nasion, and point B |
| Sagittal Analysis | ANB | ° | The angle between Nasion, point A, and point B |
| Sagittal Analysis | ANB( indl.) | ° | ANB_ind_ = (−35.16 + 0.4 · SNA + 0.2 · ML-NSL) according to Panagiotidis and Witt |
| Sagittal Analysis | SN-Ba | ° | Central saddle angle. It describes the extent of the skull base flexion |
| Sagittal Analysis | SNPg | ° | Theangle between Sella, Nasion and Pogonion |
| Sagittal Analysis | S-N | mm | The S-N line represents the anterior cranial base. It is constructed by connecting the points sella turcica and the Nasion |
| Sagittal Analysis | Go-Me | mm | The mandibular plane as a line connecting the points gonion and menton |
| Sagittal Analysis | Wits | mm | measures the extent to which the jaws are related to each other anteroposteriorly. |
| Growth Analysis | ML-NSL | ° | The angle formed between the ML and NSL lines |
| Dental Analysis | (+1/NL) | ° | The angle between upper incisors’ tooth axis and line NL |
| Dental Analysis | (+1/SN) | ° | The angle between upper incisors’ tooth axis and line SN |
| Dental Analysis | +1/NA | ° | The angle between upper incisors’ tooth axis and line NA |
| Dental Analysis | +1/NA | mm | the upper central incisor to N-A reading in millimeters provides information on the relative forward or backward positioning of the incisor teeth to the N-A line |
| Dental Analysis | -1/ML (anatomic) | ° | The relative anteroposterior angulation of the lower incisor teeth is determined by relating the most protruding incisor tooth to the mandibular plane (ML) |
| Dental Analysis | (-1/NB) | ° | The lower central incisor to N-B reading in degrees indicates the relative axial inclination of these teeth |
| Dental Analysis | (-1/NB) | mm | The lower incisor to NB line measurement in millimeters shows the relative forward or backward positioning of these teeth to the N-B line |
| Dental Analysis | Interincisal angle | ° | the inter-incisal angle relates the relative position of the upper incisor to that of the lower incisor |

Supplementary table 1.

**Supplementary Table 2A.**

|  | **Class II** | | | | | | |
| --- | --- | --- | --- | --- | --- | --- | --- |
| Variable | N | Mean | Std. Dev. | Min | Pctl. 25 | Pctl. 75 | Max |
| Age | 237 | 17 | 6.5 | 6.8 | 13 | 21 | 44 |
| 0<Age<13 | 74 (31%) |  |  |  |  |  |  |
| 14<Age<20 | 107 (45%) |  |  |  |  |  |  |
| Age>21 | 56 (24%) |  |  |  |  |  |  |
| Female | 162 (68%) |  |  |  |  |  |  |
| Male | 75 (32%) |  |  |  |  |  |  |
| NL-ML angle | 237 | 28 | 6.5 | 13 | 24 | 33 | 54 |
| NL-NSL | 237 | 8.3 | 3.6 | 1 | 6.1 | 10 | 42 |
| PFH/AFH | 237 | 65 | 5.3 | 50 | 62 | 69 | 79 |
| Gonial_angle | 237 | 128 | 7.6 | 110 | 124 | 134 | 150 |
| Facial axis | 237 | 88 | 4.6 | 70 | 85 | 91 | 102 |
| SNA angle | 237 | 86 | 50 | 75 | 80 | 86 | 847 |
| SNB angle | 237 | 75 | 5.4 | 7.8 | 73 | 78 | 84 |
| ANB angle | 237 | 7.2 | 1.8 | 3.3 | 6 | 8.3 | 16 |
| ANB_ind_ | 237 | 5.3 | 1.6 | 1.6 | 4.1 | 6.4 | 10 |
| Calculated_ANB | 237 | 2 | 0.9 | 1.1 | 1.3 | 2.6 | 5.5 |
| SN-Ba angle | 237 | 130 | 5.3 | 119 | 126 | 133 | 147 |
| SN-Pg angle | 237 | 76 | 3.5 | 65 | 74 | 79 | 85 |
| S-N (mm) | 237 | 63 | 6.9 | 30 | 59 | 65 | 87 |
| Go-Me (mm) | 237 | 59 | 5.9 | 44 | 55 | 62 | 77 |
| Wits appraisal (mm) | 237 | -1.2 | 2.5 | -9.5 | -2.8 | 0.6 | 8.3 |
| ML-NSL angle | 237 | 36 | 7.2 | 4.6 | 32 | 40 | 63 |
| +1/NL angle | 237 | 113 | 7.6 | 91 | 108 | 119 | 135 |
| +1/SNL angle | 237 | 105 | 8.3 | 78 | 99 | 110 | 124 |
| +1/NA angle | 237 | 21 | 12 | -121 | 17 | 27 | 42 |
| +1/NA (mm) | 237 | 2.9 | 2.3 | -2.8 | 1.2 | 4.4 | 9.2 |
| -1/ML (anatomic) | 237 | 97 | 9.3 | 9.5 | 92 | 102 | 120 |
| -1/NB angle | 237 | 29 | 7 | 6.8 | 25 | 33 | 50 |
| -1/NB (mm) | 237 | 5.6 | 2.6 | -0.5 | 3.8 | 7.4 | 12 |
| Dental Interincisal angle | 237 | 122 | 11 | 100 | 114 | 129 | 163 |

**Supplementary Table 2B.**

|  | **Class III** | | | | | | |
| --- | --- | --- | --- | --- | --- | --- | --- |
| Variable | N | M | Std. Dev. | Min | Pctl. 25 | Pctl. 75 | Max |
| Age | 265 | 18 | 8.1 | 6 | 13 | 21 | 54 |
| 0<Age<13 | 71 (27%) |  |  |  |  |  |  |
| 14<Age<20 | 117 (44%) |  |  |  |  |  |  |
| Age>21 | 77 (29%) |  |  |  |  |  |  |
| Female | 140 (53%) |  |  |  |  |  |  |
| Male | 125 (47%) |  |  |  |  |  |  |
| NL-ML angle | 265 | 29 | 6.9 | 12 | 24 | 33 | 55 |
| NL-NSL | 265 | 7.6 | 3.4 | -1.7 | 5.3 | 10 | 18 |
| PFH/AFH | 265 | 65 | 5.8 | 50 | 61 | 69 | 82 |
| gonialangle | 265 | 135 | 7.8 | 110 | 130 | 140 | 160 |
| Facial axis | 265 | 91 | 5.6 | 73 | 87 | 95 | 104 |
| SNA angle | 265 | 82 | 4.2 | 70 | 79 | 84 | 94 |
| SNB angle | 265 | 82 | 4.9 | 66 | 79 | 85 | 95 |
| ANB angle | 265 | -0.22 | 3 | -8.1 | -2.1 | 1.7 | 11 |
| ANB_ind_ | 265 | 4.8 | 1.6 | -0.4 | 3.8 | 5.9 | 8.6 |
| Calculated_ANB (ANB – ANB_ind_) | 265 | -5 | 2.5 | -12 | -6.6 | -3.5 | 3.2 |
| SN-Ba angle | 265 | 127 | 5.9 | 104 | 124 | 132 | 145 |
| SN-Pg angle | 265 | 82 | 5.2 | 64 | 78 | 86 | 95 |
| S-N (mm) | 265 | 61 | 6.9 | 41 | 57 | 64 | 95 |
| Go-Me (mm) | 265 | 61 | 8.6 | 41 | 56 | 66 | 100 |
| Wits appraisal (mm) | 265 | -11 | 4.5 | -25 | -14 | -8.2 | 1.2 |
| ML-NSL angle | 265 | 37 | 7.9 | 14 | 31 | 41 | 64 |
| +1/NL angle | 265 | 116 | 7.4 | 96 | 112 | 120 | 138 |
| +1/SNL angle | 265 | 107 | 12 | 10 | 102 | 114 | 135 |
| +1/NA angle | 264 | 27 | 7.5 | 7.7 | 22 | 31 | 66 |
| +1/NA (mm) | 264 | 4.4 | 2.5 | -2.6 | 2.8 | 5.8 | 13 |
| -1/ML (anatomic) | 264 | 87 | 7.8 | 62 | 82 | 92 | 105 |
| -1/NB angle | 264 | 25 | 7.1 | 3.5 | 21 | 30 | 41 |
| -1/NB (mm) | 264 | 4.9 | 2.5 | -0.8 | 2.9 | 6.5 | 12 |
| Interincisal angle | 264 | 128 | 11 | 100 | 120 | 135 | 156 |

Supplementary Table 3.

| Parameter | Groups | **diff** | **lwr** | **upr** | **p adj** |
| --- | --- | --- | --- | --- | --- |
| NL-ML angle | III_Age>21-II_14<Age<20 | 3.52 | 0.66 | 6.37 | 0.01 |
| NL-ML angle | III_Female_Age>21-II_Female_14<Age<20 | 5.18 | 0.70 | 9.65 | 0.01 |
| NL-NSL angle | III_Male-II_Female | -1.47 | -2.54 | -0.40 | 0.00 |
| PFH/AFH ratio | III_Female-II_Male | -2.44 | -4.46 | -0.42 | 0.01 |
| PFH/AFH ratio | III_Male_14<Age<20-II_Female_0<Age<13 | 3.99 | 0.29 | 7.70 | 0.02 |
| PFH/AFH ratio | III_Male_14<Age<20-II_Female_Age>21 | 3.86 | 0.13 | 7.59 | 0.03 |
| Gonial Angle | III_Female-II_Female | 7.31 | 5.01 | 9.61 | 0.00 |
| Gonial Angle | III_Male-II_Female | 5.70 | 3.33 | 8.07 | 0.00 |
| Gonial Angle | III_Female-II_Male | 6.83 | 3.98 | 9.68 | 0.00 |
| Gonial Angle | III_Male-II_Male | 5.22 | 2.31 | 8.13 | 0.00 |
| Gonial Angle | III_0<Age<13-II_0<Age<13 | 4.76 | 1.10 | 8.41 | 0.00 |
| Gonial Angle | III_14<Age<20-II_0<Age<13 | 5.01 | 1.74 | 8.27 | 0.00 |
| Gonial Angle | III_Age>21-II_0<Age<13 | 5.66 | 2.08 | 9.24 | 0.00 |
| Gonial Angle | III_0<Age<13-II_14<Age<20 | 7.76 | 4.40 | 11.13 | 0.00 |
| Gonial Angle | III_14<Age<20-II_14<Age<20 | 8.01 | 5.07 | 10.96 | 0.00 |
| Gonial Angle | III_Age>21-II_14<Age<20 | 8.66 | 5.38 | 11.95 | 0.00 |
| Gonial Angle | III_0<Age<13-II_Age>21 | 4.40 | 0.46 | 8.33 | 0.02 |
| Gonial Angle | III_14<Age<20-II_Age>21 | 4.64 | 1.07 | 8.22 | 0.00 |
| Gonial Angle | III_Age>21-II_Age>21 | 5.30 | 1.43 | 9.16 | 0.00 |
| Gonial Angle | III_Female_14<Age<20-II_Female_0<Age<13 | 5.66 | 0.77 | 10.56 | 0.01 |
| Gonial Angle | III_Female_Age>21-II_Female_0<Age<13 | 6.32 | 0.59 | 12.05 | 0.02 |
| Gonial Angle | III_Female_0<Age<13-II_Female_14<Age<20 | 9.00 | 3.92 | 14.09 | 0.00 |
| Gonial Angle | III_Female_14<Age<20-II_Female_14<Age<20 | 9.30 | 5.06 | 13.53 | 0.00 |
| Gonial Angle | III_Female_Age>21-II_Female_14<Age<20 | 9.95 | 4.78 | 15.13 | 0.00 |
| Gonial Angle | III_Male_0<Age<13-II_Female_14<Age<20 | 7.43 | 2.20 | 12.66 | 0.00 |
| Gonial Angle | III_Male_14<Age<20-II_Female_14<Age<20 | 7.40 | 2.75 | 12.04 | 0.00 |
| Gonial Angle | III_Male_Age>21-II_Female_14<Age<20 | 8.49 | 3.61 | 13.36 | 0.00 |
| Gonial Angle | III_Female_14<Age<20-II_Female_Age>21 | 5.22 | 0.29 | 10.15 | 0.03 |
| Gonial Angle | III_Female_Age>21-II_Female_Age>21 | 5.87 | 0.11 | 11.63 | 0.04 |
| Gonial Angle | III_Female_14<Age<20-II_Male_0<Age<13 | 6.01 | 0.46 | 11.55 | 0.02 |
| Gonial Angle | III_Female_Age>21-II_Male_0<Age<13 | 6.66 | 0.37 | 12.96 | 0.03 |
| Gonial Angle | III_Female_0<Age<13-II_Male_14<Age<20 | 7.37 | 1.26 | 13.48 | 0.00 |
| Gonial Angle | III_Female_14<Age<20-II_Male_14<Age<20 | 7.67 | 2.24 | 13.09 | 0.00 |
| Gonial Angle | III_Female_Age>21-II_Male_14<Age<20 | 8.32 | 2.13 | 14.51 | 0.00 |
| Gonial Angle | III_Male_14<Age<20-II_Male_14<Age<20 | 5.76 | 0.01 | 11.51 | 0.05 |
| Gonial Angle | III_Male_Age>21-II_Male_14<Age<20 | 6.85 | 0.92 | 12.79 | 0.01 |
| Facial axis | III_Female-II_Female | 2.62 | 1.08 | 4.16 | 0.00 |
| Facial axis | III_Male-II_Female | 3.58 | 1.99 | 5.17 | 0.00 |
| Facial axis | III_Female-II_Male | 2.31 | 0.40 | 4.22 | 0.01 |
| Facial axis | III_Male-II_Male | 3.27 | 1.32 | 5.22 | 0.00 |
| Facial axis | III_0<Age<13-II_0<Age<13 | 2.84 | 0.39 | 5.29 | 0.01 |
| Facial axis | III_14<Age<20-II_0<Age<13 | 2.44 | 0.25 | 4.63 | 0.02 |
| Facial axis | III_0<Age<13-II_14<Age<20 | 2.92 | 0.66 | 5.17 | 0.00 |
| Facial axis | III_14<Age<20-II_14<Age<20 | 2.52 | 0.55 | 4.49 | 0.00 |
| Facial axis | III_0<Age<13-II_Age>21 | 5.34 | 2.71 | 7.97 | 0.00 |
| Facial axis | III_14<Age<20-II_Age>21 | 4.94 | 2.55 | 7.33 | 0.00 |
| Facial axis | III_Age>21-II_Age>21 | 4.26 | 1.67 | 6.84 | 0.00 |
| Facial axis | III_Female_0<Age<13-II_Female_Age>21 | 5.41 | 1.62 | 9.19 | 0.00 |
| Facial axis | III_Female_14<Age<20-II_Female_Age>21 | 5.06 | 1.77 | 8.35 | 0.00 |
| Facial axis | III_Male_0<Age<13-II_Female_Age>21 | 5.91 | 2.03 | 9.78 | 0.00 |
| Facial axis | III_Male_14<Age<20-II_Female_Age>21 | 5.50 | 1.97 | 9.03 | 0.00 |
| Facial axis | III_Male_Age>21-II_Female_Age>21 | 5.66 | 1.99 | 9.32 | 0.00 |
| SNB angle | III_Female-II_Female | 5.49 | 3.97 | 7.02 | 0.00 |
| SNB angle | III_Male-II_Female | 7.25 | 5.67 | 8.82 | 0.00 |
| SNB angle | III_Female-II_Male | 6.05 | 4.15 | 7.94 | 0.00 |
| SNB angle | III_Male-II_Male | 7.80 | 5.87 | 9.73 | 0.00 |
| SNB angle | III_0<Age<13-II_0<Age<13 | 6.56 | 4.12 | 9.00 | 0.00 |
| SNB angle | III_14<Age<20-II_0<Age<13 | 7.71 | 5.53 | 9.89 | 0.00 |
| SNB angle | III_Age>21-II_0<Age<13 | 8.18 | 5.79 | 10.57 | 0.00 |
| SNB angle | III_0<Age<13-II_14<Age<20 | 4.56 | 2.32 | 6.81 | 0.00 |
| SNB angle | III_14<Age<20-II_14<Age<20 | 5.72 | 3.75 | 7.68 | 0.00 |
| SNB angle | III_Age>21-II_14<Age<20 | 6.18 | 3.99 | 8.38 | 0.00 |
| SNB angle | III_0<Age<13-II_Age>21 | 5.96 | 3.33 | 8.58 | 0.00 |
| SNB angle | III_14<Age<20-II_Age>21 | 7.11 | 4.72 | 9.49 | 0.00 |
| SNB angle | III_Age>21-II_Age>21 | 7.58 | 5.00 | 10.15 | 0.00 |
| SNB angle | III_Female_0<Age<13-II_Female_0<Age<13 | 5.45 | 1.70 | 9.19 | 0.00 |
| SNB angle | III_Female_14<Age<20-II_Female_0<Age<13 | 6.39 | 3.14 | 9.64 | 0.00 |
| SNB angle | III_Female_Age>21-II_Female_0<Age<13 | 6.27 | 2.47 | 10.07 | 0.00 |
| SNB angle | III_Male_0<Age<13-II_Female_0<Age<13 | 6.51 | 2.68 | 10.35 | 0.00 |
| SNB angle | III_Male_14<Age<20-II_Female_0<Age<13 | 8.12 | 4.63 | 11.60 | 0.00 |
| SNB angle | III_Male_Age>21-II_Female_0<Age<13 | 8.67 | 5.05 | 12.29 | 0.00 |
| SNB angle | III_Female_0<Age<13-II_Female_14<Age<20 | 4.05 | 0.68 | 7.42 | 0.01 |
| SNB angle | III_Female_14<Age<20-II_Female_14<Age<20 | 4.99 | 2.18 | 7.80 | 0.00 |
| SNB angle | III_Female_Age>21-II_Female_14<Age<20 | 4.87 | 1.44 | 8.31 | 0.00 |
| SNB angle | III_Male_0<Age<13-II_Female_14<Age<20 | 5.12 | 1.65 | 8.58 | 0.00 |
| SNB angle | III_Male_14<Age<20-II_Female_14<Age<20 | 6.72 | 3.63 | 9.80 | 0.00 |
| SNB angle | III_Male_Age>21-II_Female_14<Age<20 | 7.27 | 4.03 | 10.50 | 0.00 |
| SNB angle | III_Female_0<Age<13-II_Female_Age>21 | 5.56 | 1.80 | 9.32 | 0.00 |
| SNB angle | III_Female_14<Age<20-II_Female_Age>21 | 6.50 | 3.23 | 9.77 | 0.00 |
| SNB angle | III_Female_Age>21-II_Female_Age>21 | 6.38 | 2.56 | 10.20 | 0.00 |
| SNB angle | III_Male_0<Age<13-II_Female_Age>21 | 6.63 | 2.78 | 10.48 | 0.00 |
| SNB angle | III_Male_14<Age<20-II_Female_Age>21 | 8.23 | 4.72 | 11.74 | 0.00 |
| SNB angle | III_Male_Age>21-II_Female_Age>21 | 8.78 | 5.14 | 12.42 | 0.00 |
| SNB angle | III_Female_0<Age<13-II_Male_0<Age<13 | 6.93 | 2.81 | 11.05 | 0.00 |
| SNB angle | III_Female_14<Age<20-II_Male_0<Age<13 | 7.87 | 4.19 | 11.55 | 0.00 |
| SNB angle | III_Female_Age>21-II_Male_0<Age<13 | 7.75 | 3.58 | 11.93 | 0.00 |
| SNB angle | III_Male_0<Age<13-II_Male_0<Age<13 | 8.00 | 3.79 | 12.20 | 0.00 |
| SNB angle | III_Male_14<Age<20-II_Male_0<Age<13 | 9.60 | 5.71 | 13.49 | 0.00 |
| SNB angle | III_Male_Age>21-II_Male_0<Age<13 | 10.15 | 6.14 | 14.16 | 0.00 |
| SNB angle | III_Female_0<Age<13-II_Male_14<Age<20 | 4.06 | 0.01 | 8.11 | 0.05 |
| SNB angle | III_Female_14<Age<20-II_Male_14<Age<20 | 5.00 | 1.40 | 8.60 | 0.00 |
| SNB angle | III_Female_Age>21-II_Male_14<Age<20 | 4.89 | 0.78 | 8.99 | 0.01 |
| SNB angle | III_Male_0<Age<13-II_Male_14<Age<20 | 5.13 | 1.00 | 9.26 | 0.00 |
| SNB angle | III_Male_14<Age<20-II_Male_14<Age<20 | 6.73 | 2.92 | 10.54 | 0.00 |
| SNB angle | III_Male_Age>21-II_Male_14<Age<20 | 7.28 | 3.34 | 11.22 | 0.00 |
| SNB angle | III_Female_14<Age<20-II_Male_Age>21 | 6.00 | 0.92 | 11.08 | 0.01 |
| SNB angle | III_Female_Age>21-II_Male_Age>21 | 5.88 | 0.43 | 11.33 | 0.02 |
| SNB angle | III_Male_0<Age<13-II_Male_Age>21 | 6.13 | 0.66 | 11.60 | 0.01 |
| SNB angle | III_Male_14<Age<20-II_Male_Age>21 | 7.73 | 2.49 | 12.96 | 0.00 |
| SNB angle | III_Male_Age>21-II_Male_Age>21 | 8.28 | 2.95 | 13.61 | 0.00 |
| ANB angle | III_Female-II_Female | -7.23 | -7.97 | -6.49 | 0.00 |
| ANB angle | III_Male-II_Female | -7.85 | -8.62 | -7.09 | 0.00 |
| ANB angle | III_Female-II_Male | -7.00 | -7.92 | -6.09 | 0.00 |
| ANB angle | III_Male-II_Male | -7.63 | -8.57 | -6.69 | 0.00 |
| ANB angle | III_0<Age<13-II_0<Age<13 | -6.86 | -8.05 | -5.68 | 0.00 |
| ANB angle | III_14<Age<20-II_0<Age<13 | -7.50 | -8.56 | -6.44 | 0.00 |
| ANB angle | III_Age>21-II_0<Age<13 | -7.43 | -8.60 | -6.27 | 0.00 |
| ANB angle | III_0<Age<13-II_14<Age<20 | -6.97 | -8.06 | -5.88 | 0.00 |
| ANB angle | III_14<Age<20-II_14<Age<20 | -7.61 | -8.56 | -6.65 | 0.00 |
| ANB angle | III_Age>21-II_14<Age<20 | -7.54 | -8.61 | -6.47 | 0.00 |
| ANB angle | III_0<Age<13-II_Age>21 | -7.26 | -8.53 | -5.98 | 0.00 |
| ANB angle | III_14<Age<20-II_Age>21 | -7.89 | -9.05 | -6.73 | 0.00 |
| ANB angle | III_Age>21-II_Age>21 | -7.83 | -9.08 | -6.57 | 0.00 |
| ANB angle | III_Female_0<Age<13-II_Female_0<Age<13 | -6.55 | -8.38 | -4.72 | 0.00 |
| ANB angle | III_Female_14<Age<20-II_Female_0<Age<13 | -7.32 | -8.90 | -5.73 | 0.00 |
| ANB angle | III_Female_Age>21-II_Female_0<Age<13 | -6.85 | -8.71 | -4.99 | 0.00 |
| ANB angle | III_Male_0<Age<13-II_Female_0<Age<13 | -7.17 | -9.04 | -5.29 | 0.00 |
| ANB angle | III_Male_14<Age<20-II_Female_0<Age<13 | -7.71 | -9.41 | -6.01 | 0.00 |
| ANB angle | III_Male_Age>21-II_Female_0<Age<13 | -7.89 | -9.66 | -6.12 | 0.00 |
| ANB angle | III_Female_0<Age<13-II_Female_14<Age<20 | -6.77 | -8.42 | -5.13 | 0.00 |
| ANB angle | III_Female_14<Age<20-II_Female_14<Age<20 | -7.54 | -8.91 | -6.17 | 0.00 |
| ANB angle | III_Female_Age>21-II_Female_14<Age<20 | -7.07 | -8.75 | -5.40 | 0.00 |
| ANB angle | III_Male_0<Age<13-II_Female_14<Age<20 | -7.39 | -9.09 | -5.70 | 0.00 |
| ANB angle | III_Male_14<Age<20-II_Female_14<Age<20 | -7.93 | -9.44 | -6.43 | 0.00 |
| ANB angle | III_Male_Age>21-II_Female_14<Age<20 | -8.11 | -9.69 | -6.53 | 0.00 |
| ANB angle | III_Female_0<Age<13-II_Female_Age>21 | -7.02 | -8.86 | -5.18 | 0.00 |
| ANB angle | III_Female_14<Age<20-II_Female_Age>21 | -7.79 | -9.39 | -6.19 | 0.00 |
| ANB angle | III_Female_Age>21-II_Female_Age>21 | -7.32 | -9.19 | -5.46 | 0.00 |
| ANB angle | III_Male_0<Age<13-II_Female_Age>21 | -7.64 | -9.52 | -5.76 | 0.00 |
| ANB angle | III_Male_14<Age<20-II_Female_Age>21 | -8.18 | -9.90 | -6.47 | 0.00 |
| ANB angle | III_Male_Age>21-II_Female_Age>21 | -8.36 | -10.14 | -6.58 | 0.00 |
| ANB angle | III_Female_0<Age<13-II_Male_0<Age<13 | -6.60 | -8.61 | -4.58 | 0.00 |
| ANB angle | III_Female_14<Age<20-II_Male_0<Age<13 | -7.36 | -9.16 | -5.57 | 0.00 |
| ANB angle | III_Female_Age>21-II_Male_0<Age<13 | -6.90 | -8.94 | -4.86 | 0.00 |
| ANB angle | III_Male_0<Age<13-II_Male_0<Age<13 | -7.21 | -9.27 | -5.16 | 0.00 |
| ANB angle | III_Male_14<Age<20-II_Male_0<Age<13 | -7.76 | -9.66 | -5.86 | 0.00 |
| ANB angle | III_Male_Age>21-II_Male_0<Age<13 | -7.94 | -9.89 | -5.98 | 0.00 |
| ANB angle | III_Female_0<Age<13-II_Male_14<Age<20 | -6.44 | -8.42 | -4.46 | 0.00 |
| ANB angle | III_Female_14<Age<20-II_Male_14<Age<20 | -7.21 | -8.96 | -5.45 | 0.00 |
| ANB angle | III_Female_Age>21-II_Male_14<Age<20 | -6.74 | -8.74 | -4.74 | 0.00 |
| ANB angle | III_Male_0<Age<13-II_Male_14<Age<20 | -7.06 | -9.08 | -5.04 | 0.00 |
| ANB angle | III_Male_14<Age<20-II_Male_14<Age<20 | -7.60 | -9.46 | -5.74 | 0.00 |
| ANB angle | III_Male_Age>21-II_Male_14<Age<20 | -7.78 | -9.70 | -5.85 | 0.00 |
| ANB angle | III_Female_0<Age<13-II_Male_Age>21 | -6.75 | -9.40 | -4.11 | 0.00 |
| ANB angle | III_Female_14<Age<20-II_Male_Age>21 | -7.52 | -10.00 | -5.04 | 0.00 |
| ANB angle | III_Female_Age>21-II_Male_Age>21 | -7.06 | -9.72 | -4.39 | 0.00 |
| ANB angle | III_Male_0<Age<13-II_Male_Age>21 | -7.37 | -10.05 | -4.70 | 0.00 |
| ANB angle | III_Male_14<Age<20-II_Male_Age>21 | -7.92 | -10.47 | -5.36 | 0.00 |
| ANB angle | III_Male_Age>21-II_Male_Age>21 | -8.09 | -10.69 | -5.49 | 0.00 |
| ANBind | III_Male-II_Female | -0.59 | -1.08 | -0.11 | 0.01 |
| ANBind | III_0<Age<13-II_Age>21 | -1.10 | -1.90 | -0.29 | 0.00 |
| ANBind | III_14<Age<20-II_Age>21 | -0.87 | -1.60 | -0.14 | 0.01 |
| ANBind | III_Male_0<Age<13-II_Female_Age>21 | -1.23 | -2.42 | -0.04 | 0.03 |
| ANBind | III_Male_14<Age<20-II_Female_Age>21 | -1.08 | -2.17 | 0.00 | 0.05 |
| Calculated_ANB | III_Female-II_Female | -6.83 | -7.39 | -6.26 | 0.00 |
| Calculated_ANB | III_Male-II_Female | -7.26 | -7.84 | -6.67 | 0.00 |
| Calculated_ANB | III_Female-II_Male | -6.78 | -7.48 | -6.08 | 0.00 |
| Calculated_ANB | III_Male-II_Male | -7.21 | -7.93 | -6.50 | 0.00 |
| Calculated_ANB | III_0<Age<13-II_0<Age<13 | -6.35 | -7.24 | -5.46 | 0.00 |
| Calculated_ANB | III_14<Age<20-II_0<Age<13 | -7.22 | -8.02 | -6.42 | 0.00 |
| Calculated_ANB | III_Age>21-II_0<Age<13 | -7.58 | -8.46 | -6.71 | 0.00 |
| Calculated_ANB | III_0<Age<13-II_14<Age<20 | -6.28 | -7.11 | -5.46 | 0.00 |
| Calculated_ANB | III_14<Age<20-II_14<Age<20 | -7.15 | -7.87 | -6.43 | 0.00 |
| Calculated_ANB | III_Age>21-II_14<Age<20 | -7.52 | -8.32 | -6.71 | 0.00 |
| Calculated_ANB | III_0<Age<13-II_Age>21 | -6.16 | -7.12 | -5.20 | 0.00 |
| Calculated_ANB | III_14<Age<20-II_Age>21 | -7.02 | -7.90 | -6.15 | 0.00 |
| Calculated_ANB | III_Age>21-II_Age>21 | -7.39 | -8.34 | -6.45 | 0.00 |
| Calculated_ANB | III_Female_0<Age<13-II_Female_0<Age<13 | -6.05 | -7.43 | -4.68 | 0.00 |
| Calculated_ANB | III_Female_14<Age<20-II_Female_0<Age<13 | -7.09 | -8.29 | -5.90 | 0.00 |
| Calculated_ANB | III_Female_Age>21-II_Female_0<Age<13 | -7.07 | -8.47 | -5.67 | 0.00 |
| Calculated_ANB | III_Male_0<Age<13-II_Female_0<Age<13 | -6.52 | -7.93 | -5.11 | 0.00 |
| Calculated_ANB | III_Male_14<Age<20-II_Female_0<Age<13 | -7.21 | -8.50 | -5.93 | 0.00 |
| Calculated_ANB | III_Male_Age>21-II_Female_0<Age<13 | -7.88 | -9.21 | -6.54 | 0.00 |
| Calculated_ANB | III_Female_0<Age<13-II_Female_14<Age<20 | -6.15 | -7.39 | -4.90 | 0.00 |
| Calculated_ANB | III_Female_14<Age<20-II_Female_14<Age<20 | -7.18 | -8.22 | -6.15 | 0.00 |
| Calculated_ANB | III_Female_Age>21-II_Female_14<Age<20 | -7.16 | -8.43 | -5.90 | 0.00 |
| Calculated_ANB | III_Male_0<Age<13-II_Female_14<Age<20 | -6.61 | -7.89 | -5.33 | 0.00 |
| Calculated_ANB | III_Male_14<Age<20-II_Female_14<Age<20 | -7.30 | -8.44 | -6.17 | 0.00 |
| Calculated_ANB | III_Male_Age>21-II_Female_14<Age<20 | -7.97 | -9.16 | -6.77 | 0.00 |
| Calculated_ANB | III_Female_0<Age<13-II_Female_Age>21 | -5.94 | -7.33 | -4.55 | 0.00 |
| Calculated_ANB | III_Female_14<Age<20-II_Female_Age>21 | -6.98 | -8.18 | -5.77 | 0.00 |
| Calculated_ANB | III_Female_Age>21-II_Female_Age>21 | -6.96 | -8.37 | -5.55 | 0.00 |
| Calculated_ANB | III_Male_0<Age<13-II_Female_Age>21 | -6.41 | -7.82 | -4.99 | 0.00 |
| Calculated_ANB | III_Male_14<Age<20-II_Female_Age>21 | -7.10 | -8.39 | -5.81 | 0.00 |
| Calculated_ANB | III_Male_Age>21-II_Female_Age>21 | -7.76 | -9.10 | -6.42 | 0.00 |
| Calculated_ANB | III_Female_0<Age<13-II_Male_0<Age<13 | -6.23 | -7.75 | -4.71 | 0.00 |
| Calculated_ANB | III_Female_14<Age<20-II_Male_0<Age<13 | -7.27 | -8.63 | -5.92 | 0.00 |
| Calculated_ANB | III_Female_Age>21-II_Male_0<Age<13 | -7.25 | -8.79 | -5.71 | 0.00 |
| Calculated_ANB | III_Male_0<Age<13-II_Male_0<Age<13 | -6.70 | -8.25 | -5.15 | 0.00 |
| Calculated_ANB | III_Male_14<Age<20-II_Male_0<Age<13 | -7.39 | -8.83 | -5.96 | 0.00 |
| Calculated_ANB | III_Male_Age>21-II_Male_0<Age<13 | -8.05 | -9.53 | -6.58 | 0.00 |
| Calculated_ANB | III_Female_0<Age<13-II_Male_14<Age<20 | -5.86 | -7.35 | -4.37 | 0.00 |
| Calculated_ANB | III_Female_14<Age<20-II_Male_14<Age<20 | -6.90 | -8.23 | -5.58 | 0.00 |
| Calculated_ANB | III_Female_Age>21-II_Male_14<Age<20 | -6.88 | -8.39 | -5.37 | 0.00 |
| Calculated_ANB | III_Male_0<Age<13-II_Male_14<Age<20 | -6.33 | -7.85 | -4.81 | 0.00 |
| Calculated_ANB | III_Male_14<Age<20-II_Male_14<Age<20 | -7.02 | -8.43 | -5.62 | 0.00 |
| Calculated_ANB | III_Male_Age>21-II_Male_14<Age<20 | -7.68 | -9.13 | -6.23 | 0.00 |
| Calculated_ANB | III_Female_0<Age<13-II_Male_Age>21 | -5.92 | -7.91 | -3.93 | 0.00 |
| Calculated_ANB | III_Female_14<Age<20-II_Male_Age>21 | -6.96 | -8.83 | -5.09 | 0.00 |
| Calculated_ANB | III_Female_Age>21-II_Male_Age>21 | -6.94 | -8.95 | -4.93 | 0.00 |
| Calculated_ANB | III_Male_0<Age<13-II_Male_Age>21 | -6.39 | -8.40 | -4.37 | 0.00 |
| Calculated_ANB | III_Male_14<Age<20-II_Male_Age>21 | -7.08 | -9.01 | -5.15 | 0.00 |
| Calculated_ANB | III_Male_Age>21-II_Male_Age>21 | -7.74 | -9.70 | -5.78 | 0.00 |
| SN-Ba angle | III_Male-II_Female | -4.25 | -5.95 | -2.56 | 0.00 |
| SN-Ba angle | III_Male-II_Male | -3.87 | -5.95 | -1.80 | 0.00 |
| SN-Ba angle | III_14<Age<20-II_0<Age<13 | -2.46 | -4.84 | -0.08 | 0.04 |
| SN-Ba angle | III_Age>21-II_0<Age<13 | -3.84 | -6.46 | -1.23 | 0.00 |
| SN-Ba angle | III_14<Age<20-II_14<Age<20 | -2.46 | -4.61 | -0.32 | 0.01 |
| SN-Ba angle | III_Age>21-II_14<Age<20 | -3.85 | -6.25 | -1.45 | 0.00 |
| SN-Ba angle | III_Male_14<Age<20-II_Female_0<Age<13 | -5.11 | -8.87 | -1.34 | 0.00 |
| SN-Ba angle | III_Male_Age>21-II_Female_0<Age<13 | -4.97 | -8.88 | -1.05 | 0.00 |
| SN-Ba angle | III_Male_14<Age<20-II_Female_14<Age<20 | -5.14 | -8.47 | -1.80 | 0.00 |
| SN-Ba angle | III_Male_Age>21-II_Female_14<Age<20 | -5.00 | -8.49 | -1.50 | 0.00 |
| SN-Ba angle | III_Male_14<Age<20-II_Female_Age>21 | -3.95 | -7.74 | -0.16 | 0.03 |
| SN-Ba angle | III_Male_14<Age<20-II_Male_0<Age<13 | -4.74 | -8.94 | -0.53 | 0.01 |
| SN-Ba angle | III_Male_Age>21-II_Male_0<Age<13 | -4.60 | -8.93 | -0.26 | 0.03 |
| SN-Ba angle | III_Male_14<Age<20-II_Male_14<Age<20 | -4.55 | -8.67 | -0.43 | 0.02 |
| SN-Ba angle | III_Male_Age>21-II_Male_14<Age<20 | -4.41 | -8.66 | -0.15 | 0.03 |
| SN-Pg angle | III_Female-II_Female | 4.52 | 3.22 | 5.83 | 0.00 |
| SN-Pg angle | III_Male-II_Female | 6.70 | 5.35 | 8.04 | 0.00 |
| SN-Pg angle | III_Female-II_Male | 4.38 | 2.76 | 6.00 | 0.00 |
| SN-Pg angle | III_Male-II_Male | 6.56 | 4.90 | 8.21 | 0.00 |
| SN-Pg angle | III_0<Age<13-II_0<Age<13 | 4.99 | 2.89 | 7.08 | 0.00 |
| SN-Pg angle | III_14<Age<20-II_0<Age<13 | 6.17 | 4.29 | 8.04 | 0.00 |
| SN-Pg angle | III_Age>21-II_0<Age<13 | 6.77 | 4.71 | 8.82 | 0.00 |
| SN-Pg angle | III_0<Age<13-II_14<Age<20 | 3.68 | 1.75 | 5.61 | 0.00 |
| SN-Pg angle | III_14<Age<20-II_14<Age<20 | 4.86 | 3.17 | 6.55 | 0.00 |
| SN-Pg angle | III_Age>21-II_14<Age<20 | 5.46 | 3.57 | 7.34 | 0.00 |
| SN-Pg angle | III_0<Age<13-II_Age>21 | 5.28 | 3.03 | 7.54 | 0.00 |
| SN-Pg angle | III_14<Age<20-II_Age>21 | 6.46 | 4.41 | 8.51 | 0.00 |
| SN-Pg angle | III_Age>21-II_Age>21 | 7.06 | 4.85 | 9.28 | 0.00 |
| SN-Pg angle | III_Female_0<Age<13-II_Female_0<Age<13 | 4.66 | 1.46 | 7.85 | 0.00 |
| SN-Pg angle | III_Female_14<Age<20-II_Female_0<Age<13 | 5.37 | 2.60 | 8.14 | 0.00 |
| SN-Pg angle | III_Female_Age>21-II_Female_0<Age<13 | 5.43 | 2.19 | 8.67 | 0.00 |
| SN-Pg angle | III_Male_0<Age<13-II_Female_0<Age<13 | 5.76 | 2.49 | 9.03 | 0.00 |
| SN-Pg angle | III_Male_14<Age<20-II_Female_0<Age<13 | 7.74 | 4.77 | 10.71 | 0.00 |
| SN-Pg angle | III_Male_Age>21-II_Female_0<Age<13 | 8.24 | 5.16 | 11.33 | 0.00 |
| SN-Pg angle | III_Female_0<Age<13-II_Female_14<Age<20 | 3.06 | 0.19 | 5.94 | 0.03 |
| SN-Pg angle | III_Female_14<Age<20-II_Female_14<Age<20 | 3.78 | 1.38 | 6.17 | 0.00 |
| SN-Pg angle | III_Female_Age>21-II_Female_14<Age<20 | 3.84 | 0.91 | 6.77 | 0.00 |
| SN-Pg angle | III_Male_0<Age<13-II_Female_14<Age<20 | 4.17 | 1.21 | 7.12 | 0.00 |
| SN-Pg angle | III_Male_14<Age<20-II_Female_14<Age<20 | 6.15 | 3.52 | 8.78 | 0.00 |
| SN-Pg angle | III_Male_Age>21-II_Female_14<Age<20 | 6.65 | 3.89 | 9.41 | 0.00 |
| SN-Pg angle | III_Female_0<Age<13-II_Female_Age>21 | 4.90 | 1.69 | 8.11 | 0.00 |
| SN-Pg angle | III_Female_14<Age<20-II_Female_Age>21 | 5.61 | 2.83 | 8.40 | 0.00 |
| SN-Pg angle | III_Female_Age>21-II_Female_Age>21 | 5.67 | 2.42 | 8.93 | 0.00 |
| SN-Pg angle | III_Male_0<Age<13-II_Female_Age>21 | 6.00 | 2.72 | 9.29 | 0.00 |
| SN-Pg angle | III_Male_14<Age<20-II_Female_Age>21 | 7.98 | 4.99 | 10.97 | 0.00 |
| SN-Pg angle | III_Male_Age>21-II_Female_Age>21 | 8.49 | 5.38 | 11.59 | 0.00 |
| SN-Pg angle | III_Female_0<Age<13-II_Male_0<Age<13 | 4.17 | 0.65 | 7.68 | 0.01 |
| SN-Pg angle | III_Female_14<Age<20-II_Male_0<Age<13 | 4.88 | 1.75 | 8.02 | 0.00 |
| SN-Pg angle | III_Female_Age>21-II_Male_0<Age<13 | 4.94 | 1.38 | 8.50 | 0.00 |
| SN-Pg angle | III_Male_0<Age<13-II_Male_0<Age<13 | 5.27 | 1.69 | 8.86 | 0.00 |
| SN-Pg angle | III_Male_14<Age<20-II_Male_0<Age<13 | 7.25 | 3.94 | 10.57 | 0.00 |
| SN-Pg angle | III_Male_Age>21-II_Male_0<Age<13 | 7.76 | 4.34 | 11.18 | 0.00 |
| SN-Pg angle | III_Female_14<Age<20-II_Male_14<Age<20 | 4.07 | 1.01 | 7.14 | 0.00 |
| SN-Pg angle | III_Female_Age>21-II_Male_14<Age<20 | 4.13 | 0.63 | 7.63 | 0.01 |
| SN-Pg angle | III_Male_0<Age<13-II_Male_14<Age<20 | 4.46 | 0.94 | 7.99 | 0.00 |
| SN-Pg angle | III_Male_14<Age<20-II_Male_14<Age<20 | 6.44 | 3.19 | 9.70 | 0.00 |
| SN-Pg angle | III_Male_Age>21-II_Male_14<Age<20 | 6.95 | 3.59 | 10.30 | 0.00 |
| SN-Pg angle | III_Female_14<Age<20-II_Male_Age>21 | 4.99 | 0.65 | 9.32 | 0.01 |
| SN-Pg angle | III_Female_Age>21-II_Male_Age>21 | 5.04 | 0.40 | 9.69 | 0.02 |
| SN-Pg angle | III_Male_0<Age<13-II_Male_Age>21 | 5.37 | 0.71 | 10.04 | 0.01 |
| SN-Pg angle | III_Male_14<Age<20-II_Male_Age>21 | 7.36 | 2.89 | 11.82 | 0.00 |
| SN-Pg angle | III_Male_Age>21-II_Male_Age>21 | 7.86 | 3.32 | 12.40 | 0.00 |
| S-N (mm) | III_Female-II_Male | -3.72 | -6.24 | -1.19 | 0.00 |
| S-N (mm) | III_0<Age<13-II_0<Age<13 | -3.92 | -7.18 | -0.66 | 0.01 |
| S-N (mm) | III_Female_Age>21-II_Male_0<Age<13 | -5.63 | -11.18 | -0.07 | 0.04 |
| S-N (mm) | III_Male_0<Age<13-II_Male_0<Age<13 | -6.41 | -12.01 | -0.82 | 0.01 |
| Go-Me (mm) | III_Female-II_Female | 2.40 | 0.20 | 4.61 | 0.03 |
| Go-Me (mm) | III_Male-II_Female | 3.90 | 1.62 | 6.17 | 0.00 |
| Go-Me (mm) | III_Male-II_Male | 2.85 | 0.06 | 5.64 | 0.04 |
| Go-Me (mm) | III_14<Age<20-II_0<Age<13 | 4.17 | 1.06 | 7.28 | 0.00 |
| Go-Me (mm) | III_Age>21-II_0<Age<13 | 4.34 | 0.93 | 7.76 | 0.00 |
| Go-Me (mm) | III_14<Age<20-II_14<Age<20 | 3.82 | 1.01 | 6.62 | 0.00 |
| Go-Me (mm) | III_Age>21-II_14<Age<20 | 3.99 | 0.86 | 7.12 | 0.00 |
| Go-Me (mm) | III_14<Age<20-II_Age>21 | 3.44 | 0.03 | 6.84 | 0.05 |
| Go-Me (mm) | III_Male_14<Age<20-II_Female_0<Age<13 | 5.98 | 0.99 | 10.96 | 0.01 |
| Go-Me (mm) | III_Male_Age>21-II_Female_0<Age<13 | 6.45 | 1.27 | 11.63 | 0.00 |
| Go-Me (mm) | III_Male_14<Age<20-II_Female_14<Age<20 | 5.21 | 0.80 | 9.62 | 0.01 |
| Go-Me (mm) | III_Male_Age>21-II_Female_14<Age<20 | 5.69 | 1.06 | 10.31 | 0.00 |
| Go-Me (mm) | III_Male_14<Age<20-II_Female_Age>21 | 5.05 | 0.04 | 10.07 | 0.05 |
| Go-Me (mm) | III_Male_Age>21-II_Female_Age>21 | 5.53 | 0.32 | 10.74 | 0.03 |
| Wits appraisal | III_Female-II_Female | -10.07 | -11.17 | -8.97 | 0.00 |
| Wits appraisal | III_Male-II_Female | -10.50 | -11.64 | -9.37 | 0.00 |
| Wits appraisal | III_Female-II_Male | -9.91 | -11.28 | -8.55 | 0.00 |
| Wits appraisal | III_Male-II_Male | -10.35 | -11.74 | -8.95 | 0.00 |
| Wits appraisal | III_0<Age<13-II_0<Age<13 | -8.50 | -10.22 | -6.77 | 0.00 |
| Wits appraisal | III_14<Age<20-II_0<Age<13 | -10.54 | -12.08 | -8.99 | 0.00 |
| Wits appraisal | III_Age>21-II_0<Age<13 | -11.05 | -12.75 | -9.36 | 0.00 |
| Wits appraisal | III_0<Age<13-II_14<Age<20 | -8.49 | -10.09 | -6.90 | 0.00 |
| Wits appraisal | III_14<Age<20-II_14<Age<20 | -10.53 | -11.92 | -9.14 | 0.00 |
| Wits appraisal | III_Age>21-II_14<Age<20 | -11.05 | -12.60 | -9.50 | 0.00 |
| Wits appraisal | III_0<Age<13-II_Age>21 | -8.86 | -10.72 | -7.00 | 0.00 |
| Wits appraisal | III_14<Age<20-II_Age>21 | -10.90 | -12.59 | -9.21 | 0.00 |
| Wits appraisal | III_Age>21-II_Age>21 | -11.42 | -13.24 | -9.59 | 0.00 |
| Wits appraisal | III_Female_0<Age<13-II_Female_0<Age<13 | -8.48 | -11.15 | -5.82 | 0.00 |
| Wits appraisal | III_Female_14<Age<20-II_Female_0<Age<13 | -10.38 | -12.69 | -8.07 | 0.00 |
| Wits appraisal | III_Female_Age>21-II_Female_0<Age<13 | -10.08 | -12.79 | -7.37 | 0.00 |
| Wits appraisal | III_Male_0<Age<13-II_Female_0<Age<13 | -8.23 | -10.96 | -5.50 | 0.00 |
| Wits appraisal | III_Male_14<Age<20-II_Female_0<Age<13 | -10.44 | -12.92 | -7.96 | 0.00 |
| Wits appraisal | III_Male_Age>21-II_Female_0<Age<13 | -11.62 | -14.20 | -9.04 | 0.00 |
| Wits appraisal | III_Female_0<Age<13-II_Female_14<Age<20 | -8.86 | -11.26 | -6.46 | 0.00 |
| Wits appraisal | III_Female_14<Age<20-II_Female_14<Age<20 | -10.76 | -12.76 | -8.76 | 0.00 |
| Wits appraisal | III_Female_Age>21-II_Female_14<Age<20 | -10.46 | -12.90 | -8.01 | 0.00 |
| Wits appraisal | III_Male_0<Age<13-II_Female_14<Age<20 | -8.61 | -11.08 | -6.14 | 0.00 |
| Wits appraisal | III_Male_14<Age<20-II_Female_14<Age<20 | -10.81 | -13.01 | -8.62 | 0.00 |
| Wits appraisal | III_Male_Age>21-II_Female_14<Age<20 | -12.00 | -14.30 | -9.70 | 0.00 |
| Wits appraisal | III_Female_0<Age<13-II_Female_Age>21 | -8.84 | -11.51 | -6.16 | 0.00 |
| Wits appraisal | III_Female_14<Age<20-II_Female_Age>21 | -10.73 | -13.06 | -8.40 | 0.00 |
| Wits appraisal | III_Female_Age>21-II_Female_Age>21 | -10.43 | -13.15 | -7.71 | 0.00 |
| Wits appraisal | III_Male_0<Age<13-II_Female_Age>21 | -8.58 | -11.32 | -5.84 | 0.00 |
| Wits appraisal | III_Male_14<Age<20-II_Female_Age>21 | -10.79 | -13.28 | -8.29 | 0.00 |
| Wits appraisal | III_Male_Age>21-II_Female_Age>21 | -11.97 | -14.56 | -9.38 | 0.00 |
| Wits appraisal | III_Female_0<Age<13-II_Male_0<Age<13 | -8.82 | -11.75 | -5.88 | 0.00 |
| Wits appraisal | III_Female_14<Age<20-II_Male_0<Age<13 | -10.71 | -13.33 | -8.09 | 0.00 |
| Wits appraisal | III_Female_Age>21-II_Male_0<Age<13 | -10.41 | -13.38 | -7.44 | 0.00 |
| Wits appraisal | III_Male_0<Age<13-II_Male_0<Age<13 | -8.56 | -11.55 | -5.57 | 0.00 |
| Wits appraisal | III_Male_14<Age<20-II_Male_0<Age<13 | -10.77 | -13.54 | -8.00 | 0.00 |
| Wits appraisal | III_Male_Age>21-II_Male_0<Age<13 | -11.95 | -14.81 | -9.10 | 0.00 |
| Wits appraisal | III_Female_0<Age<13-II_Male_14<Age<20 | -8.04 | -10.92 | -5.15 | 0.00 |
| Wits appraisal | III_Female_14<Age<20-II_Male_14<Age<20 | -9.93 | -12.49 | -7.37 | 0.00 |
| Wits appraisal | III_Female_Age>21-II_Male_14<Age<20 | -9.63 | -12.55 | -6.71 | 0.00 |
| Wits appraisal | III_Male_0<Age<13-II_Male_14<Age<20 | -7.78 | -10.72 | -4.84 | 0.00 |
| Wits appraisal | III_Male_14<Age<20-II_Male_14<Age<20 | -9.99 | -12.70 | -7.27 | 0.00 |
| Wits appraisal | III_Male_Age>21-II_Male_14<Age<20 | -11.17 | -13.97 | -8.37 | 0.00 |
| Wits appraisal | III_Female_0<Age<13-II_Male_Age>21 | -9.47 | -13.32 | -5.61 | 0.00 |
| Wits appraisal | III_Female_14<Age<20-II_Male_Age>21 | -11.36 | -14.98 | -7.74 | 0.00 |
| Wits appraisal | III_Female_Age>21-II_Male_Age>21 | -11.06 | -14.94 | -7.18 | 0.00 |
| Wits appraisal | III_Male_0<Age<13-II_Male_Age>21 | -9.21 | -13.11 | -5.32 | 0.00 |
| Wits appraisal | III_Male_14<Age<20-II_Male_Age>21 | -11.42 | -15.14 | -7.69 | 0.00 |
| Wits appraisal | III_Male_Age>21-II_Male_Age>21 | -12.60 | -16.39 | -8.81 | 0.00 |
| ML-NSL angle | III_Female_Age>21-II_Female_14<Age<20 | 5.20 | 0.20 | 10.20 | 0.03 |
| +1/NL angle | III_Male-II_Female | 3.60 | 1.30 | 5.90 | 0.00 |
| +1/NL angle | III_Female-II_Male | 3.10 | 0.34 | 5.86 | 0.02 |
| +1/NL angle | III_Male-II_Male | 5.01 | 2.19 | 7.83 | 0.00 |
| +1/NL angle | III_14<Age<20-II_14<Age<20 | 4.55 | 1.72 | 7.38 | 0.00 |
| +1/NL angle | III_Age>21-II_14<Age<20 | 5.29 | 2.13 | 8.45 | 0.00 |
| +1/NL angle | III_14<Age<20-II_Age>21 | 4.70 | 1.26 | 8.13 | 0.00 |
| +1/NL angle | III_Age>21-II_Age>21 | 5.44 | 1.73 | 9.15 | 0.00 |
| +1/NL angle | III_Male_Age>21-II_Female_14<Age<20 | 6.21 | 1.56 | 10.87 | 0.00 |
| +1/NL angle | III_Male_Age>21-II_Female_Age>21 | 6.37 | 1.13 | 11.61 | 0.00 |
| +1/NL angle | III_Female_14<Age<20-II_Male_14<Age<20 | 5.97 | 0.79 | 11.15 | 0.01 |
| +1/NL angle | III_Male_14<Age<20-II_Male_14<Age<20 | 6.93 | 1.44 | 12.42 | 0.00 |
| +1/NL angle | III_Male_Age>21-II_Male_14<Age<20 | 8.82 | 3.15 | 14.48 | 0.00 |
| +1/NL angle | III_Male_14<Age<20-II_Male_Age>21 | 7.78 | 0.25 | 15.31 | 0.04 |
| +1/NL angle | III_Male_Age>21-II_Male_Age>21 | 9.66 | 2.00 | 17.33 | 0.00 |
| +1/SNL angle | III_Male-II_Female | 4.07 | 0.90 | 7.24 | 0.01 |
| +1/SNL angle | III_Male-II_Male | 5.08 | 1.20 | 8.97 | 0.00 |
| +1/SNL angle | III_14<Age<20-II_14<Age<20 | 4.68 | 0.75 | 8.60 | 0.01 |
| +1/SNL angle | III_Age>21-II_14<Age<20 | 4.83 | 0.45 | 9.22 | 0.02 |
| +1/SNL angle | III_14<Age<20-II_Age>21 | 5.21 | 0.44 | 9.98 | 0.02 |
| +1/SNL angle | III_Age>21-II_Age>21 | 5.37 | 0.21 | 10.52 | 0.04 |
| +1/SNL angle | III_Male_Age>21-II_Female_14<Age<20 | 7.35 | 0.91 | 13.79 | 0.01 |
| +1/SNL angle | III_Male_Age>21-II_Female_Age>21 | 8.25 | 1.00 | 15.51 | 0.01 |
| +1/SNL angle | III_Male_14<Age<20-II_Male_14<Age<20 | 7.87 | 0.27 | 15.47 | 0.03 |
| +1/SNL angle | III_Male_Age>21-II_Male_14<Age<20 | 10.00 | 2.15 | 17.84 | 0.00 |
| +1/NA angle | III_Female-II_Female | 3.41 | 0.45 | 6.38 | 0.02 |
| +1/NA angle | III_Male-II_Female | 5.64 | 2.59 | 8.69 | 0.00 |
| +1/NA angle | III_Female-II_Male | 6.30 | 2.63 | 9.97 | 0.00 |
| +1/NA angle | III_Male-II_Male | 8.53 | 4.78 | 12.27 | 0.00 |
| +1/NA angle | III_0<Age<13-II_14<Age<20 | 5.00 | 0.68 | 9.31 | 0.01 |
| +1/NA angle | III_14<Age<20-II_14<Age<20 | 7.95 | 4.18 | 11.72 | 0.00 |
| +1/NA angle | III_Age>21-II_14<Age<20 | 9.00 | 4.77 | 13.22 | 0.00 |
| +1/NA angle | III_14<Age<20-II_Age>21 | 6.15 | 1.57 | 10.72 | 0.00 |
| +1/NA angle | III_Age>21-II_Age>21 | 7.20 | 2.24 | 12.16 | 0.00 |
| +1/NA angle | III_Female_14<Age<20-II_Female_14<Age<20 | 5.62 | 0.25 | 10.99 | 0.03 |
| +1/NA angle | III_Male_14<Age<20-II_Female_14<Age<20 | 6.70 | 0.81 | 12.59 | 0.01 |
| +1/NA angle | III_Male_Age>21-II_Female_14<Age<20 | 8.91 | 2.73 | 15.09 | 0.00 |
| +1/NA angle | III_Male_Age>21-II_Female_Age>21 | 8.49 | 1.54 | 15.45 | 0.00 |
| +1/NA angle | II_Male_14<Age<20-II_Male_0<Age<13 | -9.27 | -17.42 | -1.12 | 0.01 |
| +1/NA angle | III_Female_0<Age<13-II_Male_14<Age<20 | 8.44 | 0.69 | 16.18 | 0.02 |
| +1/NA angle | III_Female_14<Age<20-II_Male_14<Age<20 | 11.89 | 5.02 | 18.77 | 0.00 |
| +1/NA angle | III_Female_Age>21-II_Male_14<Age<20 | 11.19 | 3.30 | 19.09 | 0.00 |
| +1/NA angle | III_Male_0<Age<13-II_Male_14<Age<20 | 10.44 | 2.54 | 18.34 | 0.00 |
| +1/NA angle | III_Male_14<Age<20-II_Male_14<Age<20 | 12.98 | 5.69 | 20.27 | 0.00 |
| +1/NA angle | III_Male_Age>21-II_Male_14<Age<20 | 15.18 | 7.66 | 22.71 | 0.00 |
| +1/NA angle | III_Male_Age>21-II_Male_Age>21 | 10.59 | 0.41 | 20.77 | 0.03 |
| +1/NA (mm) | III_Female-II_Female | 1.29 | 0.57 | 2.01 | 0.00 |
| +1/NA (mm) | III_Male-II_Female | 1.83 | 1.09 | 2.57 | 0.00 |
| +1/NA (mm) | III_Female-II_Male | 1.44 | 0.54 | 2.33 | 0.00 |
| +1/NA (mm) | III_Male-II_Male | 1.97 | 1.06 | 2.89 | 0.00 |
| +1/NA (mm) | III_Age>21-II_0<Age<13 | 1.41 | 0.31 | 2.51 | 0.00 |
| +1/NA (mm) | III_0<Age<13-II_14<Age<20 | 1.09 | 0.06 | 2.13 | 0.03 |
| +1/NA (mm) | III_14<Age<20-II_14<Age<20 | 2.22 | 1.32 | 3.13 | 0.00 |
| +1/NA (mm) | III_Age>21-II_14<Age<20 | 2.68 | 1.67 | 3.70 | 0.00 |
| +1/NA (mm) | III_14<Age<20-II_Age>21 | 1.96 | 0.86 | 3.05 | 0.00 |
| +1/NA (mm) | III_Age>21-II_Age>21 | 2.42 | 1.23 | 3.61 | 0.00 |
| +1/NA (mm) | III_Male_Age>21-II_Female_0<Age<13 | 1.68 | 0.01 | 3.35 | 0.05 |
| +1/NA (mm) | III_Female_14<Age<20-II_Female_14<Age<20 | 2.02 | 0.73 | 3.32 | 0.00 |
| +1/NA (mm) | III_Female_Age>21-II_Female_14<Age<20 | 2.22 | 0.62 | 3.82 | 0.00 |
| +1/NA (mm) | III_Male_14<Age<20-II_Female_14<Age<20 | 2.32 | 0.89 | 3.74 | 0.00 |
| +1/NA (mm) | III_Male_Age>21-II_Female_14<Age<20 | 2.92 | 1.43 | 4.42 | 0.00 |
| +1/NA (mm) | III_Female_14<Age<20-II_Female_Age>21 | 1.67 | 0.16 | 3.19 | 0.02 |
| +1/NA (mm) | III_Female_Age>21-II_Female_Age>21 | 1.87 | 0.09 | 3.65 | 0.03 |
| +1/NA (mm) | III_Male_14<Age<20-II_Female_Age>21 | 1.96 | 0.34 | 3.59 | 0.00 |
| +1/NA (mm) | III_Male_Age>21-II_Female_Age>21 | 2.57 | 0.89 | 4.25 | 0.00 |
| +1/NA (mm) | III_Female_14<Age<20-II_Male_14<Age<20 | 2.28 | 0.62 | 3.94 | 0.00 |
| +1/NA (mm) | III_Female_Age>21-II_Male_14<Age<20 | 2.48 | 0.56 | 4.39 | 0.00 |
| +1/NA (mm) | III_Male_14<Age<20-II_Male_14<Age<20 | 2.57 | 0.81 | 4.33 | 0.00 |
| +1/NA (mm) | III_Male_Age>21-II_Male_14<Age<20 | 3.18 | 1.36 | 5.00 | 0.00 |
| +1/NA (mm) | III_Female_14<Age<20-II_Male_Age>21 | 2.37 | 0.02 | 4.72 | 0.05 |
| +1/NA (mm) | III_Female_Age>21-II_Male_Age>21 | 2.57 | 0.04 | 5.10 | 0.04 |
| +1/NA (mm) | III_Male_14<Age<20-II_Male_Age>21 | 2.66 | 0.24 | 5.08 | 0.02 |
| +1/NA (mm) | III_Male_Age>21-II_Male_Age>21 | 3.27 | 0.81 | 5.73 | 0.00 |
| -1/ML | III_Female-II_Female | -10.58 | -13.12 | -8.03 | 0.00 |
| -1/ML | III_Male-II_Female | -9.35 | -11.96 | -6.73 | 0.00 |
| -1/ML | III_Female-II_Male | -10.25 | -13.40 | -7.10 | 0.00 |
| -1/ML | III_Male-II_Male | -9.02 | -12.23 | -5.81 | 0.00 |
| -1/ML | III_0<Age<13-II_0<Age<13 | -8.66 | -12.71 | -4.62 | 0.00 |
| -1/ML | III_14<Age<20-II_0<Age<13 | -9.56 | -13.18 | -5.94 | 0.00 |
| -1/ML | III_Age>21-II_0<Age<13 | -8.28 | -12.26 | -4.31 | 0.00 |
| -1/ML | III_0<Age<13-II_14<Age<20 | -10.87 | -14.60 | -7.15 | 0.00 |
| -1/ML | III_14<Age<20-II_14<Age<20 | -11.77 | -15.03 | -8.51 | 0.00 |
| -1/ML | III_Age>21-II_14<Age<20 | -10.50 | -14.15 | -6.84 | 0.00 |
| -1/ML | III_0<Age<13-II_Age>21 | -8.41 | -12.77 | -4.06 | 0.00 |
| -1/ML | III_14<Age<20-II_Age>21 | -9.31 | -13.27 | -5.35 | 0.00 |
| -1/ML | III_Age>21-II_Age>21 | -8.04 | -12.33 | -3.75 | 0.00 |
| -1/ML | III_Female_0<Age<13-II_Female_0<Age<13 | -7.35 | -13.55 | -1.15 | 0.01 |
| -1/ML | III_Female_14<Age<20-II_Female_0<Age<13 | -8.03 | -13.41 | -2.65 | 0.00 |
| -1/ML | III_Female_Age>21-II_Female_0<Age<13 | -7.17 | -13.51 | -0.82 | 0.01 |
| -1/ML | III_Male_14<Age<20-II_Female_0<Age<13 | -7.15 | -12.92 | -1.38 | 0.00 |
| -1/ML | III_Female_0<Age<13-II_Female_14<Age<20 | -12.68 | -18.27 | -7.10 | 0.00 |
| -1/ML | III_Female_14<Age<20-II_Female_14<Age<20 | -13.37 | -18.02 | -8.71 | 0.00 |
| -1/ML | III_Female_Age>21-II_Female_14<Age<20 | -12.50 | -18.25 | -6.76 | 0.00 |
| -1/ML | III_Male_0<Age<13-II_Female_14<Age<20 | -11.47 | -17.22 | -5.73 | 0.00 |
| -1/ML | III_Male_14<Age<20-II_Female_14<Age<20 | -12.49 | -17.59 | -7.38 | 0.00 |
| -1/ML | III_Male_Age>21-II_Female_14<Age<20 | -11.09 | -16.45 | -5.74 | 0.00 |
| -1/ML | III_Female_0<Age<13-II_Female_Age>21 | -9.11 | -15.34 | -2.88 | 0.00 |
| -1/ML | III_Female_14<Age<20-II_Female_Age>21 | -9.79 | -15.21 | -4.38 | 0.00 |
| -1/ML | III_Female_Age>21-II_Female_Age>21 | -8.93 | -15.31 | -2.55 | 0.00 |
| -1/ML | III_Male_0<Age<13-II_Female_Age>21 | -7.90 | -14.28 | -1.52 | 0.00 |
| -1/ML | III_Male_14<Age<20-II_Female_Age>21 | -8.92 | -14.72 | -3.11 | 0.00 |
| -1/ML | III_Male_Age>21-II_Female_Age>21 | -7.52 | -13.55 | -1.49 | 0.00 |
| -1/ML | III_Female_0<Age<13-II_Male_0<Age<13 | -12.02 | -18.85 | -5.19 | 0.00 |
| -1/ML | III_Female_14<Age<20-II_Male_0<Age<13 | -12.71 | -18.80 | -6.62 | 0.00 |
| -1/ML | III_Female_Age>21-II_Male_0<Age<13 | -11.85 | -18.81 | -4.88 | 0.00 |
| -1/ML | III_Male_0<Age<13-II_Male_0<Age<13 | -10.81 | -17.78 | -3.85 | 0.00 |
| -1/ML | III_Male_14<Age<20-II_Male_0<Age<13 | -11.83 | -18.27 | -5.39 | 0.00 |
| -1/ML | III_Male_Age>21-II_Male_0<Age<13 | -10.43 | -17.08 | -3.79 | 0.00 |
| -1/ML | III_Female_0<Age<13-II_Male_14<Age<20 | -8.58 | -15.29 | -1.87 | 0.00 |
| -1/ML | III_Female_14<Age<20-II_Male_14<Age<20 | -9.26 | -15.22 | -3.30 | 0.00 |
| -1/ML | III_Female_Age>21-II_Male_14<Age<20 | -8.40 | -15.24 | -1.55 | 0.00 |
| -1/ML | III_Male_0<Age<13-II_Male_14<Age<20 | -7.37 | -14.21 | -0.52 | 0.02 |
| -1/ML | III_Male_14<Age<20-II_Male_14<Age<20 | -8.38 | -14.70 | -2.06 | 0.00 |
| -1/ML | III_Male_Age>21-II_Male_14<Age<20 | -6.99 | -13.51 | -0.46 | 0.02 |
| -1/ML | III_Female_14<Age<20-II_Male_Age>21 | -9.30 | -17.71 | -0.88 | 0.02 |
| -1/NB angle | III_Female-II_Female | -3.90 | -6.02 | -1.79 | 0.00 |
| -1/NB angle | III_Male-II_Female | -3.76 | -5.94 | -1.59 | 0.00 |
| -1/NB angle | III_Female-II_Male | -3.95 | -6.57 | -1.33 | 0.00 |
| -1/NB angle | III_Male-II_Male | -3.81 | -6.48 | -1.14 | 0.00 |
| -1/NB angle | III_0<Age<13-II_0<Age<13 | -3.53 | -6.86 | -0.19 | 0.03 |
| -1/NB angle | III_0<Age<13-II_14<Age<20 | -5.67 | -8.75 | -2.60 | 0.00 |
| -1/NB angle | III_14<Age<20-II_14<Age<20 | -4.95 | -7.64 | -2.26 | 0.00 |
| -1/NB angle | III_0<Age<13-II_Age>21 | -5.58 | -9.18 | -1.99 | 0.00 |
| -1/NB angle | III_14<Age<20-II_Age>21 | -4.87 | -8.13 | -1.60 | 0.00 |
| -1/NB angle | III_Female_0<Age<13-II_Female_14<Age<20 | -6.36 | -11.00 | -1.73 | 0.00 |
| -1/NB angle | III_Female_14<Age<20-II_Female_14<Age<20 | -4.70 | -8.56 | -0.84 | 0.00 |
| -1/NB angle | III_Male_0<Age<13-II_Female_14<Age<20 | -5.42 | -10.19 | -0.65 | 0.01 |
| -1/NB angle | III_Male_14<Age<20-II_Female_14<Age<20 | -5.88 | -10.12 | -1.64 | 0.00 |
| -1/NB angle | III_Female_0<Age<13-II_Female_Age>21 | -6.52 | -11.70 | -1.35 | 0.00 |
| -1/NB angle | III_Female_14<Age<20-II_Female_Age>21 | -4.86 | -9.35 | -0.36 | 0.02 |
| -1/NB angle | III_Male_0<Age<13-II_Female_Age>21 | -5.58 | -10.87 | -0.29 | 0.03 |
| -1/NB angle | III_Male_14<Age<20-II_Female_Age>21 | -6.04 | -10.86 | -1.22 | 0.00 |
| -1/NB angle | III_Female_0<Age<13-II_Male_0<Age<13 | -5.82 | -11.48 | -0.15 | 0.04 |
| -1/NB (mm) | III_0<Age<13-II_0<Age<13 | -1.25 | -2.42 | -0.07 | 0.03 |
| -1/NB (mm) | III_0<Age<13-II_14<Age<20 | -1.11 | -2.20 | -0.02 | 0.04 |
| -1/NB (mm) | III_0<Age<13-II_Age>21 | -1.78 | -3.05 | -0.51 | 0.00 |
| -1/NB (mm) | III_14<Age<20-II_Age>21 | -1.35 | -2.50 | -0.20 | 0.01 |
| -1/NB (mm) | III_Female_0<Age<13-II_Female_Age>21 | -1.90 | -3.72 | -0.08 | 0.03 |
| -1/NB (mm) | III_Female_0<Age<13-II_Male_0<Age<13 | -2.16 | -4.15 | -0.16 | 0.02 |
| -1/NB (mm) | III_Male_0<Age<13-II_Male_0<Age<13 | -2.07 | -4.11 | -0.04 | 0.04 |
| Interincisal angle | III_Female-II_Female | 7.06 | 3.75 | 10.37 | 0.00 |
| Interincisal angle | III_Male-II_Female | 5.93 | 2.52 | 9.34 | 0.00 |
| Interincisal angle | III_Female-II_Male | 5.69 | 1.59 | 9.80 | 0.00 |
| Interincisal angle | III_Male-II_Male | 4.56 | 0.38 | 8.75 | 0.03 |
| Interincisal angle | III_0<Age<13-II_0<Age<13 | 10.12 | 4.88 | 15.35 | 0.00 |
| Interincisal angle | III_14<Age<20-II_0<Age<13 | 7.36 | 2.68 | 12.04 | 0.00 |
| Interincisal angle | III_0<Age<13-II_14<Age<20 | 8.37 | 3.55 | 13.19 | 0.00 |
| Interincisal angle | III_14<Age<20-II_14<Age<20 | 5.61 | 1.40 | 9.83 | 0.00 |
| Interincisal angle | III_0<Age<13-II_Age>21 | 9.00 | 3.37 | 14.63 | 0.00 |
| Interincisal angle | III_14<Age<20-II_Age>21 | 6.24 | 1.12 | 11.36 | 0.01 |
| Interincisal angle | III_Female_0<Age<13-II_Female_0<Age<13 | 9.82 | 1.76 | 17.88 | 0.00 |
| Interincisal angle | III_Female_0<Age<13-II_Female_14<Age<20 | 10.48 | 3.23 | 17.74 | 0.00 |
| Interincisal angle | III_Female_14<Age<20-II_Female_14<Age<20 | 6.47 | 0.42 | 12.52 | 0.02 |
| Interincisal angle | III_Male_0<Age<13-II_Female_14<Age<20 | 8.49 | 1.02 | 15.96 | 0.01 |
| Interincisal angle | III_Male_14<Age<20-II_Female_14<Age<20 | 7.19 | 0.55 | 13.82 | 0.02 |
| Interincisal angle | III_Female_0<Age<13-II_Female_Age>21 | 11.05 | 2.95 | 19.15 | 0.00 |
| Interincisal angle | III_Female_14<Age<20-II_Female_Age>21 | 7.04 | 0.00 | 14.07 | 0.05 |
| Interincisal angle | III_Male_0<Age<13-II_Female_Age>21 | 9.06 | 0.77 | 17.35 | 0.02 |
| Interincisal angle | III_Male_14<Age<20-II_Female_Age>21 | 7.75 | 0.20 | 15.30 | 0.04 |
| Interincisal angle | III_Female_0<Age<13-II_Male_0<Age<13 | 12.91 | 4.03 | 21.78 | 0.00 |
| Interincisal angle | III_Female_14<Age<20-II_Male_0<Age<13 | 8.90 | 0.98 | 16.81 | 0.01 |
| Interincisal angle | III_Male_0<Age<13-II_Male_0<Age<13 | 10.92 | 1.87 | 19.96 | 0.00 |
| Interincisal angle | III_Male_14<Age<20-II_Male_0<Age<13 | 9.61 | 1.24 | 17.99 | 0.01 |

**Supplementary Figure 1.**


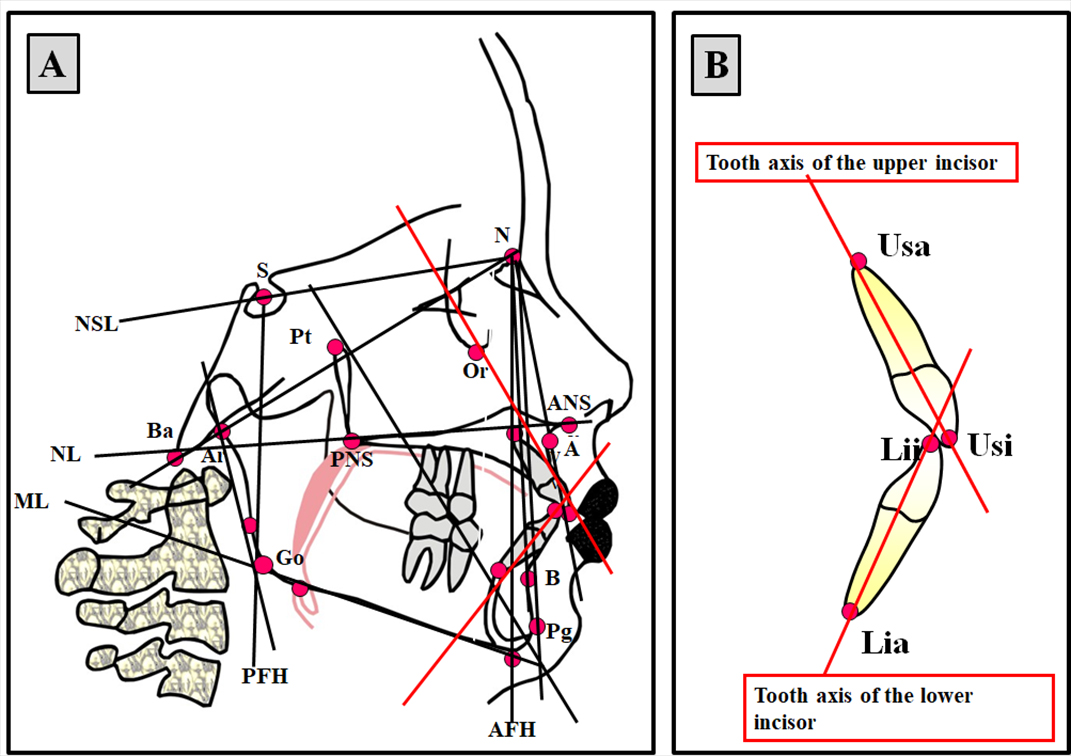


**Supplementary Figure 2A.**


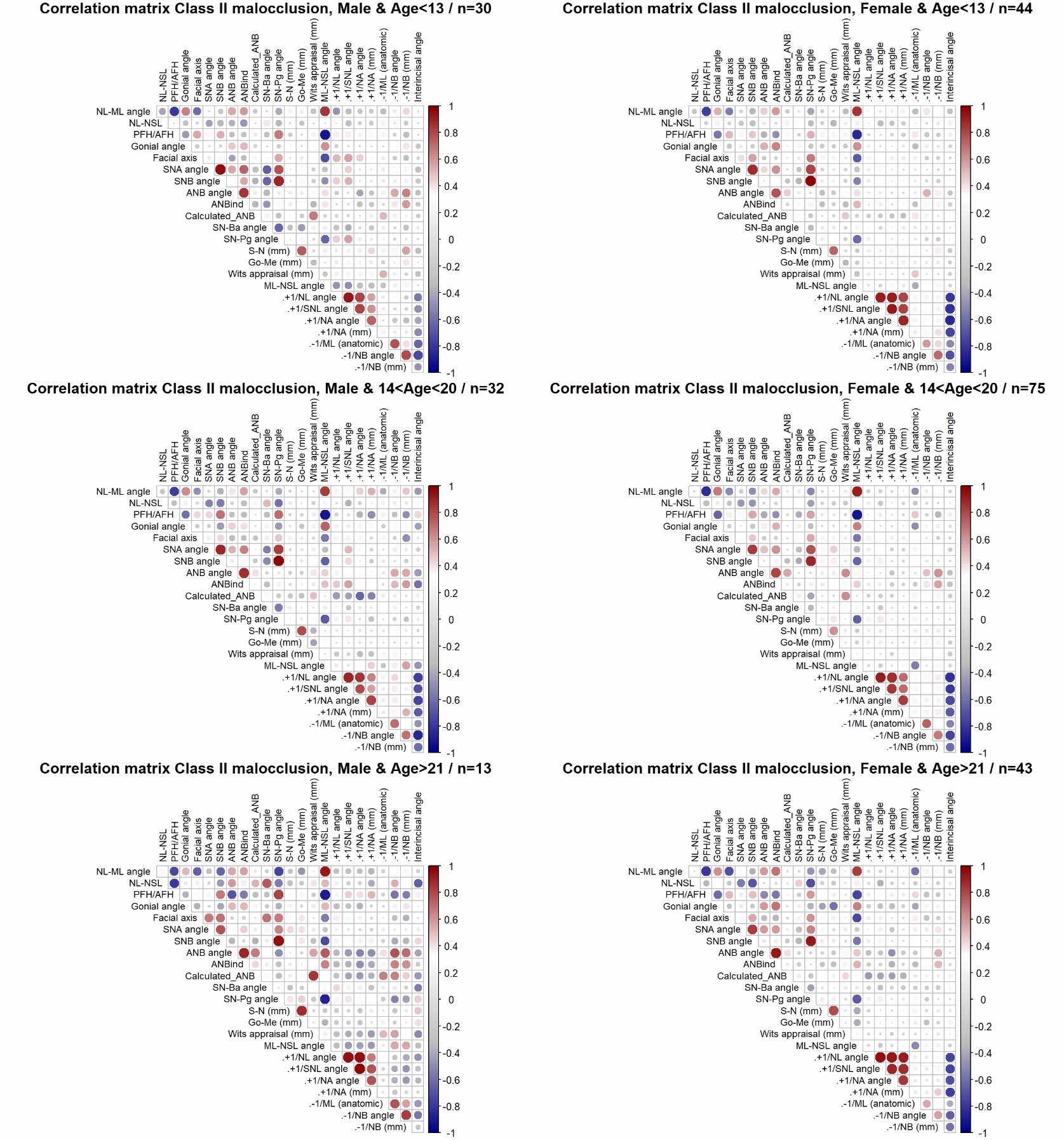


**Supplementary**
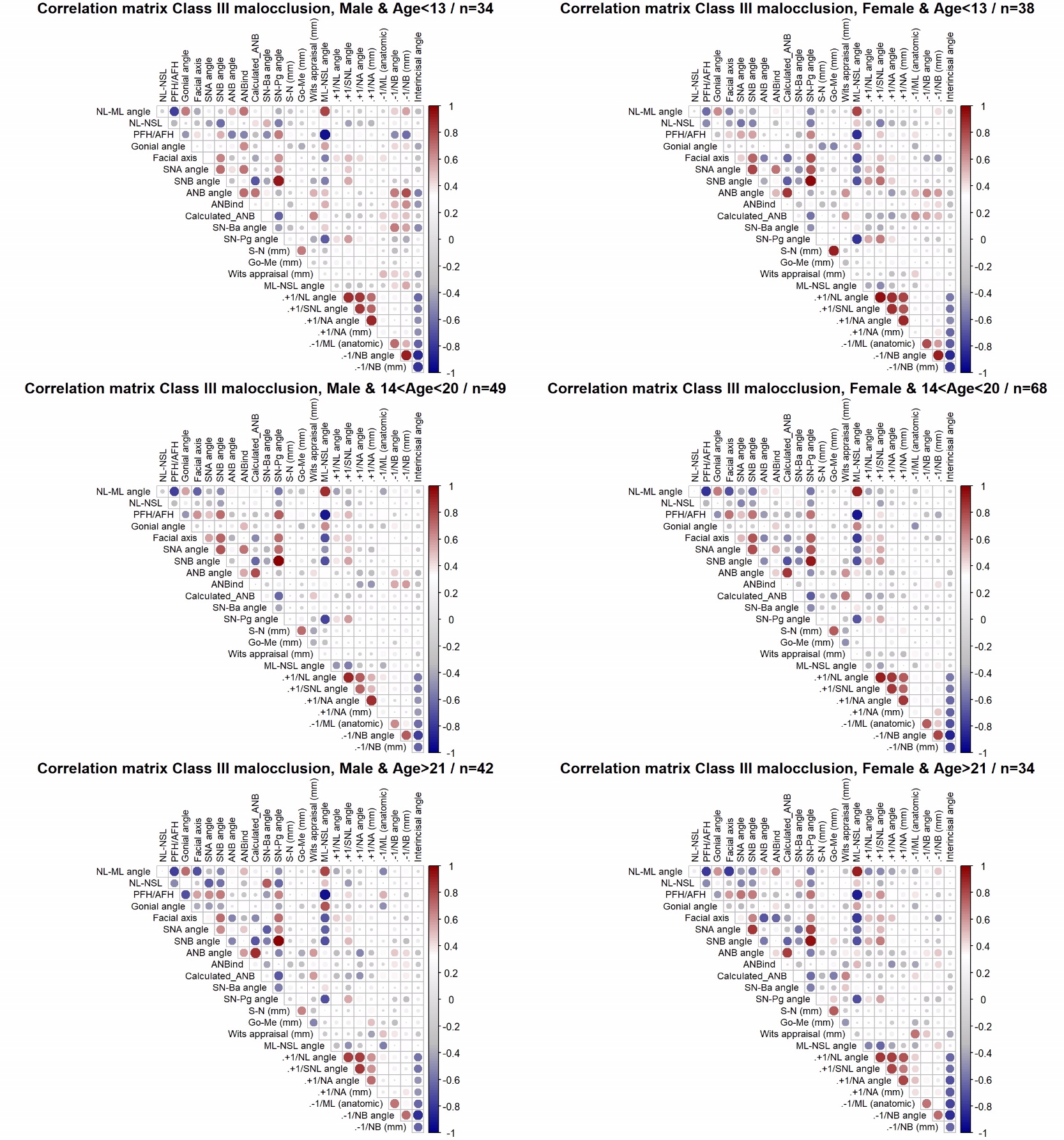
**Figure 2B.**
