# Supplementary material for: Lateral cephalometric parameters among Arab skeletal classes II and III patients and applying machine learning models
Source: Clin Oral Investig. 2024 Sep 3;28(9):511. doi: 10.1007/s00784-024-05900-2 (PMC11369042; doi:10.1007/s00784-024-05900-2)
Supplement: Supplementary file 2 — Supplementary Material 2 [file 784_2024_5900_MOESM2_ESM.docx]

**Supplementary Table 1.** Relevant cephalometric variables were analyzed, including their definition and position in **Supplementary** Fig. 1 for angular measurements.

**Supplementary Table 2.** Detailed information about SCIIMO patients. (N)- sample size, (M)-Mean, (Std. Dev.)- Standard Deviation, (Min)- Minimum Value, (Pctl. 25)- 25th percentile, (Pctl. 75)- 75th percentile, (Max)-Maximum Value (**2A**), while **Supplementary** Table 2B shows the information about the tested SCIIIMO patients. (N)- sample size, (M)-Mean, (Std. Dev.)- Standard Deviation, (Min)- Minimum Value, (Pctl. 25)- 25th percentile, (Pctl. 75)- 75th percentile, (Max)-Maximum Value.

**Supplementary Table 3.** Multiple groups comparisons of cephalometric parameters using the Tukey method. Significant differences are indicated by p-values less than 0.01 and 0.05. This table shows the significant differences when comparing different skeletal classes and subgroups of gender and age.

**Supplementary Fig. 1.** Representation of the most crucial points extracted from the lateral cephalogram in this study (**1A**). Among these points are Nasion (N), Sella (S), Pterygoid point (Pt), Basion (Ba), Orbitale (Or), Subspinale (“A” Point), Supramental (“B” point), and other points. **Supplementary Fig. 1B** shows the tooth axis of the upper and lower incisors to determine their positions and angles.

**Supplementary Fig. 2.** The heatmaps present the Spearman correlation between different cephalometric parameters for both SCIIMO and SCIIIMO patients. Color coding signifies the strength and direction of the correlation: blue indicates a negative correlation (strongest at ρ = -1), red indicates a positive correlation (strongest at ρ = 1), and the intensity of the color reflects the correlation strength. **Supplementary** Figs. 2A and 2B present correlations for SCIIMO and SCIIIMO patients, respectively, further differentiated by gender and age groups.
